# Wavelength-optimized Two-Photon Polymerization Using Initiators Based on Multipolar Aminostyryl-1,3,5-triazines

**DOI:** 10.1038/s41598-018-35301-x

**Published:** 2018-11-22

**Authors:** Maximilian Tromayer, Peter Gruber, Arnulf Rosspeintner, Aliasghar Ajami, Wolfgang Husinsky, Felix Plasser, Leticia González, Eric Vauthey, Aleksandr Ovsianikov, Robert Liska

**Affiliations:** 1 Institute of Applied Synthetic Chemistry, TU Wien (Technische Universitaet Wien), Getreidemarkt 9/163/MC, 1060 Vienna, Austria; 2 Institute of Materials Science and Technology, TU Wien (Technische Universitaet Wien), Getreidemarkt 9/308, 1060 Vienna, Austria; 3 Department of Physical Chemistry, University of Geneva, 30 Quai Ernest Ansermet, CH-1211 Geneva 4, Switzerland; 4 Faculty of Physics, Semnan University, 35131-19111 Semnan, Iran; 5 Institute of Applied Physics, TU Wien (Technische Universitaet Wien), Wiedner Hauptstrasse 8-10/134, 1040 Vienna, Austria; 6 Institute for Theoretical Chemistry, Faculty of Chemistry, University of Vienna, Waehringerstrasse 17, 1090 Vienna, Austria; 7 Austrian Cluster for Tissue Regeneration (www.tissue-regeneration.at), Vienna, Austria

## Abstract

Two-photon induced polymerization (2PP) based 3D printing is a powerful microfabrication tool. Specialized two-photon initiators (2PIs) are critical components of the employed photosensitive polymerizable formulations. This work investigates the cooperative enhancement of two-photon absorption cross sections (σ_2PA_) in a series of 1,3,5-triazine-derivatives bearing 1-3 aminostyryl-donor arms, creating dipolar, quadrupolar and octupolar push-pull systems. The multipolar 2PIs were successfully prepared and characterized, σ_2PA_ were determined using z-scan at 800 nm as well as spectrally resolved two-photon excited fluorescence measurements, and the results were compared to high-level ab initio computations. Modern tunable femtosecond lasers allow 2PP-processing at optimum wavelengths tailored to the absorption behavior of the 2PI. 2PP structuring tests revealed that while performance at 800 nm is similar, at their respective σ_2PA_-maxima the octupolar triazine-derivative outperforms a well-established ketone-based quadrupolar reference 2PI, with significantly lower fabrication threshold at exceedingly high writing speeds up to 200 mm/s and a broader window for ideal processing parameters.

## Introduction

In recent years, the process of two-photon induced polymerization (2PP) has attracted considerable interest because it enables true 3D printing with a resolution in the sub-micrometer range^1^. Parts containing ultra-small features like photonic crystals^2^, optical waveguides^3^, microelectronic components^4^ and scaffolds for tissue engineering^5,6^ may thus be produced. The properties of the photoinitiator (PI) used to induce the radical photopolymerization are crucial for obtaining high quality microstructures, as well as achieving high writing speeds, broad processing windows and low polymerization thresholds, which all are necessary preconditions for industrial application of 2PP-processing. Commercial PIs used in UV-light induced one-photon polymerization of classical coatings can be employed to generate microstructures via 2PP^7,8^. However, they suffer from limitations such as very narrow windows for ideal processing parameters and exceedingly slow achievable writing speeds due to their inefficient two-photon absorption (2PA), expressed as a low 2PA cross section (σ_2PA_)^9^. Thus it is desirable to investigate compounds with high σ_2PA_ regarding their suitability as specialized, highly efficient two-photon initiators (2PIs).

Investigations of the relationship between molecular structure and σ_2PA_ found that essential factors promoting a high σ_2PA_ are an extended π-conjugated core of good co-planarity, bearing various electronic donor and/or acceptor groups that introduce low-energy excitations with large transition and/or mesomeric dipole moments^1,10^. Besides dipolar push-pull-systems, quadrupolar, octupolar and more generally branched/multipolar 2PA chromophores (even dendritic and polymeric 2PA active compounds) are favorable as electronic coupling between the branches leads to a cooperative enhancement that can cause a rise of σ_2PA_^10^.

**Figure 1:**
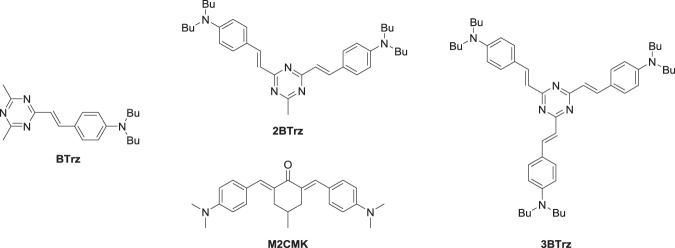
Two-photon initiators discussed in this work: dipolar **BTrz**, quadrupolar **2BTrz** and reference **M2CMK**, octupolar **3BTrz**.

The heterocyclic system 1,3,5-triazine is strongly electron deficient, so it is an excellent electronic acceptor group, and it has three carbon atoms to which branching substituents can be attached to create dipolar-, quadrupolar-, and octupolar push-pull-systems. Detailed structural analyses of star shaped 1,3,5-triazine-based compounds were carried out to investigate the planarity of the system. X-ray structure analyses of the one- and two-branched counterparts and a computer model of the three-branched compound revealed a highly planar conformation of the π-systems^11^. Furthermore, derivatives of heterocycles that contain pyridine-type nitrogen atoms often exhibit low fluorescence quantum yields^12^. This is desirable for 2PIs as fluorescence is a common loss channel, competing with the initiation of polymerization. The recently developed ketone-based 2PI **M2CMK** (Fig. 1) takes advantage of carbonyl moieties as electronic acceptor groups that ensure low fluorescence and is used in this work as a reference material^12,13^. The 2PA-behavior of 1,3,5-triazine derivatives has been investigated before; however, their application as 2PIs, especially regarding high writing speeds up to over 100 mm/s, has not been demonstrated so far. Previous studies show that 1,3,5-triazine derivatives bearing 1,4-phenylenevinylene arms with terminal dialkylamino-groups (aminostyryl-1,3,5-triazines) exhibit relatively high 2PA that strongly increases in a non-linear fashion with the number of branches^11,14,15^. These factors make branched systems containing a triazine core element promising 2PI-candidates, so that in this work a series of multipolar aminostyryl-1,3,5-triazines (Fig. 1) was prepared to investigate their relevant photophysical properties and particularly their efficiency regarding the application as 2PIs.

## Results

### Synthesis

The molecules **BTrz**, **2BTrz**, and **3BTrz** (Fig. 1) based on the 1,3,5-triazine acceptor form a series of di-, quadru-, and octupolar molecules, respectively, that were synthesized to investigate their suitability as 2PIs. Previous investigations of similar series regarding their 2PA behaviour employed methyl- and ethyl-groups as substituents on the terminal amino-groups^14,15^. As high solubility of 2PIs in polymerizable resin mixtures is crucial for the 2PP process, dibutylamino-groups were used as solubility promoting electronic donor groups in this work to circumvent the limited solubility often associated with highly planarized aromatic π-systems.

**Figure 2:**
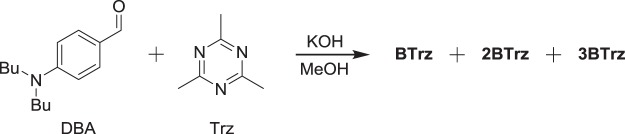
Synthesis of **BTrz**, **2BTrz**, and **3BTrz** from 4-(dibutylamino)benzaldehyde (DBA) and 2,4,6-trimethyl-1,3,5-triazine (Trz).

The required precursor 2,4,6-trimethyl-1,3,5-triazine (Trz) was prepared according to literature^16^ from commercially available ethyl acetimidate hydrochloride, which is first converted to the free base and then trimerized under the influence of catalytic amounts of glacial acetic acid. The three increasingly branched aminostyryl triazine derivatives were then obtained analogous to literature^14^ by subsequently reacting the three methyl groups of Trz with 4-(dibutylamino)benzaldehyde (DBA) in a Knoevenagel-type condensation under alkaline catalysis using potassium hydroxide in methanol (Fig. 2).

For **BTrz** only rather low yields of around 10% could be obtained even with a molar ratio of 2:1 for Trz:DBA. This is presumably because **BTrz**, due to additional resonance stabilization of intermediate carbanions, is more reactive than Trz towards condensation reactions on the methyl groups. Thus considerable amounts of **2BTrz** as well as intermediate highly polar addition products are formed despite the unfavorable molar ratios of the starting materials.

**2BTrz** and **3BTrz** were isolated in yields of 62% and 23% respectively from a reaction mixture with a molar ratio of 1:3.5 for Trz:DBA.

### Photophysical properties

The basic relevant one- and two-photon photophysical properties of **BTrz**, **2BTrz**, and **3BTrz** as well as the reference **M2CMK** in THF are summarized in Table 1.

**Table 1:** Photophysical properties for 1PA processes of **BTrz**, **2BTrz**, **3BTrz**, and the reference **M2CMK**.

2PI	λ^1PA^_abs_ [nm]	λ^1PA^_em_ [nm]	Φ^1PA^_em_	ε (λ^1PA^_abs_) [10^4^ M^−1^cm^−1^]	σ_2PA_^z-scan^ [GM]	σ_2PA_^2PEF^ [GM]
**BTrz**	412	490	0.055	4.3	60	175 (800 nm)/175 (815 nm)
**2BTrz**	432	510	0.20	8.0	244	440 (800 nm)
**3BTrz**	434	510	0.20	12.0	275	370 (800 nm)/510 (750 nm)
**M2CMK**	425	510	0.003	4.7	191^13^	500 (800 nm)/800 (760 nm)

λ^1PA^_abs_, λ^1PA^_em_, and Φ^1PA^_em_ in THF. The values of σ_2PA_ are given at 796 nm for the z-scan technique, and at 800 nm as well as the respective 2PA maximum for two-photon excited fluorescence (2PEF) measurements.

#### One Photon Spectra

**Figure 3:**
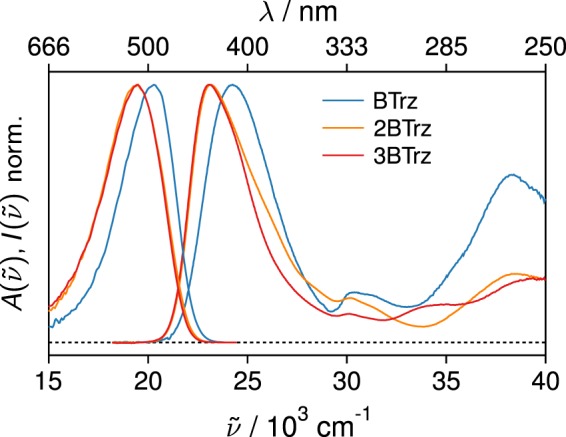
Normalized absorption and emission spectra of **BTrz** (blue), **2BTrz** (orange), and **3BTrz** (red) in tetrahydrofuran.

Figure 3 shows the electronic absorption and emission spectra of **BTrz**, **2BTrz** and **3BTrz** in THF. All three samples show a pronounced lowest energy absorption band slightly above 400 nm. The extinction coefficient of this band scales with the number of branches and the values are in good agreement with those published for a series of similar compounds^11^. The lowest energy absorption band of **2BTrz** and **3BTrz** is slightly shifted to lower energies (by 1200 cm^−1^) with respect to **BTrz**. In addition, this band shows a pronounced shoulder at higher energy for **2BTrz**, which is absent for **3BTrz**. These findings are in line with previous reports on a similar series of di-, quadru- and octupolar donor-acceptor systems^17^ and can be similarly rationalized using a Frenkel exciton model for the interactions among the branches. All three samples exhibit structureless emission spectra with a Stokes shift of almost 4000 cm^−1^, indicating significant charge-transfer character in the excited state. The emission quantum yields for **2BTrz** and **3BTrz** amount to 0.2, thus being by almost a factor of 4 and 100 larger than those for **BTrz** and **M2CMK**, respectively. Whether the nonradiative deactivation processes, accounting for the major portion of the population decay, are due to internal conversion back to the ground state, or intersystem crossing to energetically accessible triplet states, will be discussed in a forthcoming publication.

#### Two-photon absorption cross sections, σ_2PA_

**Figure 4:**
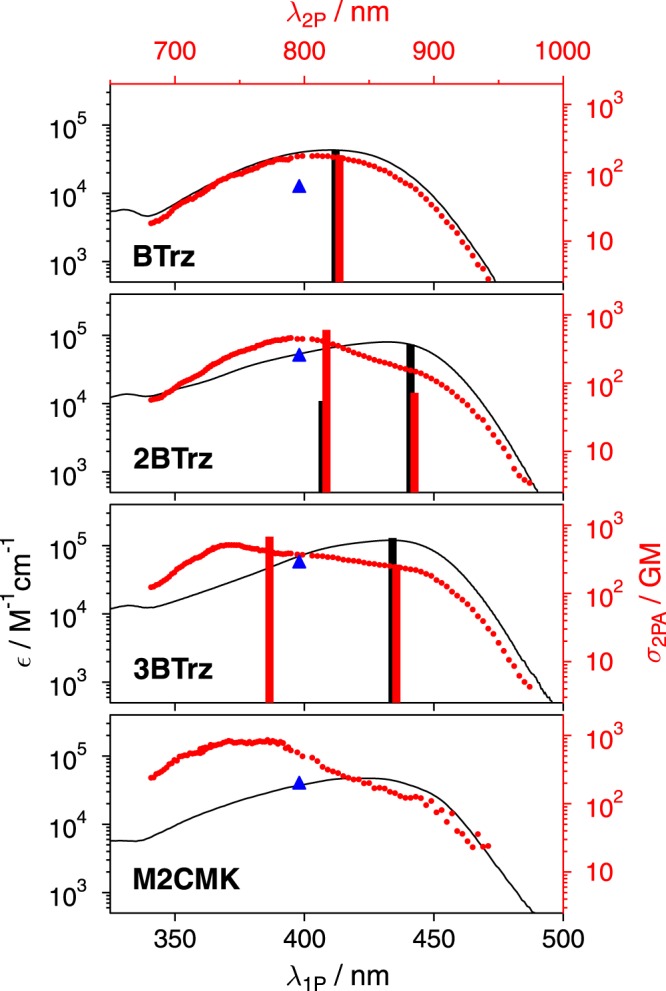
1PA, (ε, black line), and 2PA cross-section spectra σ_2PA_ (red dots) of **BTrz**, **2BTrz**, **3BTrz**, and **M2CMK** in THF. 2PA cross-sections determined via the z-scan at 796 nm are shown as blue triangles. Calculated one-photon (black columns) and two-photon allowed transition wavelengths (red columns), are shown as stick-spectra (degenerate or near-degenerate transitions are presented as a single transition). All calculated results (f and σ_2PA_ from Table 2) were red-shifted by 3500 cm^−1^ and scaled with a single constant factor to match the experimental data for **BTrz**. Note that the 2PA stick spectra in black were offset by 6 nm from the 1PA peaks for clarity of presentation.

A strong non-linear dependence on the number of branches was observed for the σ_2PA_ measured by z-scan at 796 nm. Doubling the number of branches from one branch in **BTrz** (60 GM) to two branches in **2BTrz** (244 GM) leads to a 4.1-fold cross section for the latter compound. However, adding a third branch in **3BTrz** (275 GM) only leads to a slight increase (4.6-fold the value of **BTrz**) of the cross section compared to **2BTrz** (Fig. 4).

**Figure 5:**
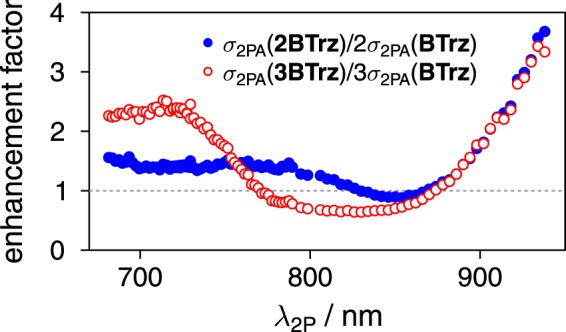
Wavelength dependence of the branching effect on the relative enhancement of the two-photon cross section of **2BTrz** (●) and **3BTrz** (o) with respect to **BTrz**.

A qualitatively similar picture is obtained from two-photon excited fluorescence (2PEF) measurements, which allow for monitoring a large spectral range of technical interest (680 to 1000 nm). The fact that the σ_2PA_ values at 796 nm obtained from the two different methods differ slightly (cf. Table 1), may be attributed to the difference in sample concentration of almost 3 orders of magnitude, as required by the different methods. In addition, it is well known that absorption based techniques may be prone to complications like e.g. disturbing excited state absorption, etc^18,19^. Fluorescence based techniques on the other hand are prone to larger errors, when the fluorescence quantum yields are very small (as e.g. for M2CMK)^20^. Fig. 4 shows a comparison of the one- and two-photon cross sections as a function of wavelength. For dipolar **BTrz** both spectra are virtually identical in terms of bandshape, with the maximum close to 800 nm. The situation completely changes for **2BTrz** and **3BTrz**, which both show strong two-photon maxima at significantly lower wavelengths than the one-photon maxima. While for **2BTrz** this maximum exactly shows up at 800 nm, it is shifted by almost 50 nm to the blue for **3BTrz**. Thus, whereas the two-photon cross section maxima scale with increasing branch number as expected from calculations (vide infra), the cross section at 800 nm is almost the same for the two multipolar samples. Figure 5 shows the relative (i.e. normalized with respect to the number of branches) enhancement of **2BTrz** and **3BTrz** with respect to **BTrz**. In line with literature^17^, a significant enhancement for quadrupolar **2BTrz** and octupolar **3BTrz** is observed in the red part of the spectrum. The enhancement of octupolar **3BTrz** in the blue part of the spectrum is significantly more pronounced than that for quadrupolar **B2Trz**. Contrary to the results from the aforementioned refs^3,17^, **BTrz** shows an “enhancement” below 1 for the spectral range where **BTrz** has its maximum.

#### Computational investigation

The molecular structures of the methyl analogues of the molecules considered here (**MTrz**, **2MTrz**, **3MTrz**), were optimized in THF solution using density functional theory (DFT) and the M06-2X functional^21^. The resulting structures are almost perfectly planar, suggesting strong π-conjugation effects along the whole molecule connecting the amino groups to the triazine ring. Excitation energies, as well as one- and two-photon absorption strengths, computed using the ab-initio ACD(2) method^22,23^ in THF solution, are presented in Table 2. The data reflects the experimental results of Table 1 well, with the exception that the absorption maxima are consistently shifted to somewhat higher energies (by approximately 3500 cm^−1^). In the case of **MTrz**, the lowest excited state possesses delocalized charge transfer (CT) character in which electron density shifts from the amino group through the side chain into the triazine ring. In the case of **2MTrz** (**3MTrz**) two (three) such states are present, one for each side chain. While the interactions of these states could be quantified by the use of essential state models^24,25^, here we will discuss the spectra on a qualitative level. In the case of **2MTrz**, two states with non-vanishing one-photon absorption are computed, in agreement with the fact that a shoulder of the main peak is seen in the absorption spectrum at around 27000 cm^−1^ (Fig. 3). The lower energy state possesses the stronger one-photon absorption strength. According to a three-state essential state model^25^, this state then serves as an intermediate state for the enhanced two-photon absorption of the higher energy state. In the case of **3MTrz** the situation is somewhat more involved. In this case, approximate *C*_*3h*_ symmetry is present and the states transform as the irreducible representations of this point group. Accordingly, there are two intense quasi-degenerate states of E’ symmetry and a third A’ state, which is one-photon forbidden. This combination leads to only one peak in the linear absorption spectrum, in agreement with Fig. 3. In analogy to **2MTrz** the dark state possesses the largest two-photon absorption strength. In summary, the results show that moving from **MTrz** to **2MTrz** qualitatively changes the mode of two-photon absorption resulting in an increase by more than a factor of three, while only a smaller modification occurs when proceeding to **3MTrz**.

**Table 2:** Computational investigation of the lowest singlet excited states of **MTrz, 2MTrz, 3MTrz**: Vertical excitation wavelengths (λ, nm), oscillator strengths for one-photon absorption (f) and two-photon absorption crossections (σ_2PA_, given in millions of atomic units).

2PI	λ	f	σ_2PA_
**MTrz**	360	1.09	2.40
**2MTrz**	382/356	1.89/0.28	0.96/8.00
**3MTrz**	377/374/339	1.66/1.66/0.00	1.77/1.75/9.00

### 2PP structuring tests

In literature, several methods have been used to quantitatively test 2PI performance. Single-line writing is well suited to test resolution limits^26^, while printing of more complex shapes at different laser intensities and writing speeds gives a more detailed and practically relevant picture regarding the performance of 2PP as a 3D microfabrication technique. Since the absorption maxima of 2PIs can only be roughly controlled by chromphore design and tunable fs-pulsed NIR-laser systems have been commercially available for some time^27^, in this work the influence of adjusting the laser wavelength to the σ_2PA_-maxima of the 2PIs (Table 1 and Fig. 4) was also investigated.

Thus arrays of defined woodpile-structures were produced varying the laser power (4–22 mW for low energy segment and 50–140 mW for high energy segment) and writing speed (20–200 mm/s) at 800 nm (the wavelength most commonly used with commercial Ti:sapphire fs-lasers) as well as the laser wavelength corresponding to the σ_2PA_-maxima, using formulations containing the 2PIs in a mixture of acrylate resins (ETA/TTA = 1:1). To take into account the different size of the delocalized π-systems and maintain comparability, **2BTrz** and the reference **M2CMK** were dissolved at 5 µmol 2PI/g resin, while **BTrz** was used at 2-fold and **3BTrz** at 0.66-fold concentration. While the crystalline compounds **3BTrz** and **M2CMK** required the addition of acetone as a co-solvent for dissolution (removed in vacuo before processing), **BTrz** and **2BTrz** were dissolved far easier by the acrylates mixture.

**Figure 6:**
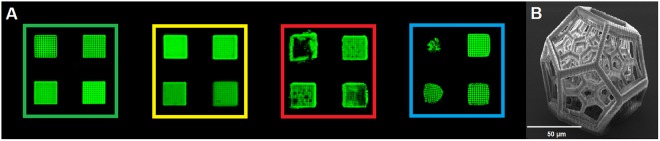
(**A**) Quality classes of woodpile structures (LSM images), their respective color codes and visual examples, (**B**) Complex 3D structure printed with **2BTrz** (SEM image).

Laser scanning microscopy (LSM) images of the polymerized woodpile-structures were judged visually and divided in four different quality classes (Fig. 6A), indicating excellent structures with fine hatch-lines in green, good structures with thicker hatch-lines and/or small defects in yellow, structures of inacceptable quality due to holes and exploded regions caused by overexposure in red, and structures that were distorted, incomplete or collapsed due to underexposure in blue. For 2PP 3D printing applications, ideal processing windows (green and yellow color codes) over a broad range of parameters are desired, as well as low laser power fabrication thresholds that allow splitting of the initial laser beam for parallel processing at high feed rates for high throughput in mass production, and high laser power overexposure thresholds for thermally induced decomposition of the material^28^.

Figure 6B highlights the virtues of 2PP as a 3D printing technique and demonstrates that structures with complex geometry, delicate features and overhangs can be produced using the novel triazine 2PI **2BTrz** at impressive writing speeds up to 200 mm/s.

**Figure 7:**
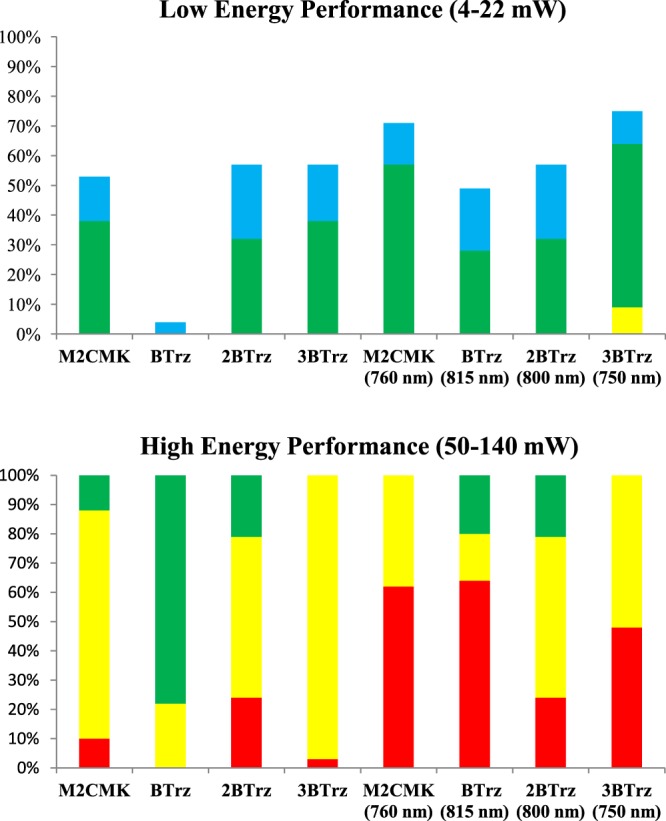
Percentage of structures in the different quality classes (color code corresponds to Fig. 6). Low energy performance of 2PIs was evaluated by printing of woodpiles from 4–22 mW laser power and high energy performance from 50–140 mW, each at writing speeds from 20 to 200 mm/s.

Figure 7 visualizes the processing windows of the 2PIs. The data represent the percentage of structures falling into a certain quality class that were obtained by varying writing speeds from 20 to 200 mm/s in 20 mm/s steps, and laser powers from 4–22 mW in 2 mW steps to assess low energy performance and from 50–140 mW in 10 mW steps to assess high energy performance respectively. Table 3 lists the individual threshold laser powers for the fabrication of green class structures at the highest tested writing speed, as well as first red class (overexposed) structures at the lowest tested speed. At 800 nm, **3BTrz** has a processing window similar to reference **M2CMK**, with a 20 mW higher overexposure threshold, but also more yellow class structures. Compared to the latter, **2BTrz** (that is already at its 2PA maximum at 800 nm) has a smaller ideal processing window, manifesting in a slightly higher fabrication threshold as well as a lower overexposure threshold. Despite the adjustment of 2PI concentration to compensate the reduced size of the π-system, **BTrz** apparently produces significantly fewer radicals compared to the other 2PIs, so that no ideal structures could be obtained in the low energy segment, and no overexposure was observed in the high energy segment. Even though only a 15 nm increase of the laser wavelength was required to print at the 2PA maximum of **BTrz**, the overall performance was most drastically affected by the wavelength shift compared to the other 2PIs. At the same time, the ideal processing window at the 2PA maximum was the smallest of the tested 2PIs. Both **3BTrz** and **M2CMK** produce radicals more efficiently at their respective 2PA maxima of 750 nm and 760 nm, resulting in both lower fabrication and overexposure thresholds. Overall, at its 2PA maximum **3BTrz** has the broadest ideal processing window of all tested compounds, with a fabrication threshold of 12 mW (14 mW for **M2CMK** at 760 nm) and an overexposure threshold of 120 mW (100 mW for **M2CMK** at 760 nm).

**Table 3:** Laser power in mW for the fabrication thresholds (first green class structure at 200 mm/s) and overexposure thresholds (first red class structure at 20 mm/s) of the different 2PIs.

2PI	fabrication threshold (800 nm)	fabrication threshold (σ_2PA_-maximum)	overexposure threshold (800 nm)	overexposure threshold (σ_2PA_-maximum)
**BTrz**	—	20	—	60
**2BTrz**	20	20	70	70
**3BTrz**	18	12	120	60
**M2CMK**	18	14	100	60

Thresholds of **BTrz** at 800 nm were outside the tested range.

## Discussion

A series of multipolar aminostyryl-1,3,5-triazine two-photon initiators (2PIs) with one (dipolar), two (quadrupolar), and three (octupolar) styryl donor arms, **BTrz**, **2BTrz**, and **3BTrz**, was successfully prepared and evaluated in comparison to the well-established quadrupolar benzylidene ketone based reference **M2CMK**. While the one-photon absorption and emission maxima were similar both within the triazine series and compared to the reference, the fluorescence quantum yields of the triazine series were 1-2 orders of magnitude larger than for the ketone based system. The triazines displayed high two-photon absorption cross sections (σ_2PA_) similar to the reference 2PI, both in z-scan and two-photon excited fluorescence (2PEF) based measurements, and a marked cooperative enhancement of **2BTrz** compared to the dipolar analogue that flattened again for the octupolar system. This was also in accordance with computations performed in this study. The use of dibutylamino-donor groups ensured excellent solubility and processability of the 2PIs in the acrylate-based resin used for two-photon polymerization (2PP) based 3D printing. At 800 nm, a common operation wavelength for 2PP printers employing commercial fs-pulsed Ti:sapphire lasers, the ideal processing windows and overall performance of **2BTrz** and **3BTrz** were similar to the reference **M2CMK**, while **BTrz** required much higher laser powers to be active, even though the 2PI concentration was adjusted to account for the smaller delocalized π-system. By tuning the laser wavelength to the σ_2PA_-maxima determined via 2PEF-spectroscopy, all investigated systems showed a marked increase in sensitivity, i.e. thresholds for fabrication of ideal structures as well as thermally induced decomposition of the formulation were lowered. Interestingly, and despite a significantly lower absolute value of σ_2PA_ at its maximum compared to **M2CMK**, **3BTrz** exhibited a larger ideal processing window at the optimum wavelength. Thus, the study demonstrated that besides the choice and design of the employed 2PI, optimizing the laser wavelength is a valuable step to increase efficiency and performance of 2PP based 3D printing.

## Methods

### Materials

Ethyl acetimidate hydrochloride and 4-(dibutylamino)benzaldehyde (DBA) were purchased from Sigma-Aldrich and used without further purification. The solvents and other reagents were purchased from Sigma Aldrich, Fluka and Merck and were dried and purified by standard laboratory methods. Trimethylolpropane triacrylate (TTA, Genomer 1330) and ethoxylated-(20/3)-trimethylolpropane triacrylate (ETA, Sartomer 415) were received as a gift from Rahn and Sartomer, respectively. Thin layer chromatography analysis was performed on silica gel 60 F_254_ aluminium sheets from Merck. Column chromatography was performed by preparative MPLC using a Buechi Sepacore Flash System (Buechi pump module C-605, Buechi control unit C-620, Buechi UV-Photometer C-635 with peak detection set at λ = 295 nm, Buechi fraction collector C-660). Glass and polyethylene columns were used, packed with Silicagel 60 on VWR silica gel 60 (0.040–0.063 mm particle size).

The preparation and analysis of the photosensitive compounds and formulations was conducted in an orange light lab. The windows and fluorescent lamps were covered in adhesive foils or coated so that light with a wavelength < 520 nm was cut off.

### Synthesis

*2,4,6-Trimethyl-1,3,5-triazine (Trz)*. Prepared according to literature^16^. Colorless crystals, yield 50%. mp: 54–56 °C, ^1^H NMR (200 MHz, CDCl_3_) δ (ppm) = 2.46 (9 H, *s*),^13^C-NMR (50 MHz, CDCl_3_) δ (ppm) = 175.8, 25.3.

*4-[(1E)-2-(4,6-Dimethyl-1,3,5-triazin-2-yl)ethenyl]-N,N-dibutylbenzenamine (****BTrz****)*. All steps were performed in an orange light room. To Trz (677 mg, 5.50 mmol, 2.0 eq.) dissolved in a 20% w/w solution of KOH in MeOH (10 mL), a solution of DBA (642 mg, 2.75 mmol, 1.0 eq.) in MeOH (10 mL) was added. The reaction mixture magnetically stirred under argon for 48 h at 60 °C. After cooling to room temperature, the reaction mixture was dissolved in DCM (150 mL), washed with saturated NH_4_Cl-solution (3 × 50 mL) and deionized water (100 mL). The organic phase was dried with Na_2_SO_4_ and stripped of solvents in vacuo. The product was isolated via column chromatography using Toluene:Et_2_O = 75:25 as eluent. Bright yellow oil **BTrz**, 10% yield. TLC: R_f_ = 0.25 (Tol/Et_2_O 3:1). ^1^H NMR (200 MHz, CDCl_3_) δ (ppm) = 8.13 (1 H, *d*, J = 15.7 Hz), 7.50 (2 H, *d*, J = 8.8 Hz), 6.81 (1 H, *d*, J = 15.7 Hz), 6.63 (2 H, *d*, J = 8.8 Hz), 3.32 (4 H, *t*, J = 7.8 Hz), 2.61 (6 H, *s*), 1.51–1.69 (4 H, *m*), 1.13–1.41 (4 H, *m*), 0.97 (6 H, *t*, J = 7.2 Hz). ^13^C-NMR (50 MHz, CDCl_3_) δ (ppm) = 175.5, 171.7, 149.6, 143.0, 130.2, 122.2, 119.3, 111.3, 50.8, 29.4, 25.7, 20.3, 14.0. HR-MS m/z: [M + H]^+^ calculated for C_21_H_30_N_4_ 339.2543; found 339.2561.

*4,4′-[(6-Methyl-1,3,5-triazine-2,4-diyl)di-(1E)-2,1-ethenediyl]bis[N,N-dibutylbenzenamine] (****2BTrz****)*. All steps were performed in an orange light room. To Trz (185 mg, 1.50 mmol, 1.0 eq.) dissolved in a 20% w/w solution of KOH in MeOH (10 mL), a solution of DBA (1.23 g, 5.25 mmol, 3.5 eq.) in MeOH (10 mL) was added. The reaction mixture magnetically stirred under argon for 120 h at 60 °C. Workup analogous to **BTrz**, using Toluene:Et_2_O = 85:15 as eluent for column chromatography. **2BTrz** elutes after **3BTrz** and residual DBA. Bright orange oil **2BTrz**, 62% yield. TLC: R_f_ = 0.55 (Tol/Et_2_O 85:15). ^1^H NMR (200 MHz, CDCl_3_) δ (ppm) = 8.16 (2 H, *d*, J = 15.8 Hz), 7.53 (4 H, *d*), 6.86 (2 H, *d*, J = 15.7 Hz), 6.64 (4 H, *d*, J = 8.8 Hz), 3.32 (8 H, *t*, J = 7.50 Hz), 2.63 (3 H, *s*), 1.50–1.71 (8 H, *m*), 1.38 (8 H, *qd*, *J* = 7.2 and 14.7 Hz), 0.98 (12 H, *t*, J = 7.20 Hz). ^13^C-NMR (50 MHz, CDCl_3_) δ (ppm) = 175.2, 171.5, 149.5, 142.1, 130.0, 122.5, 120.1, 111.3, 50.8, 29.5, 25.9, 20.3, 14.0. HR-MS m/z: [M + 2 H]^2+^ calculated for C_36_H_51_N_5_ 277.7145; found 277.7137.

*4,4′,4″*-*[1,3,5-Triazine-2,4,6-triyltri-(1E)-2,1-ethenediyl]tris[N,N-dibutylbenzenamine] (****3BTrz****)*. Synthesis and workup analogous to **2BTrz**, using Toluene:Et_2_O = 95:5 as eluent for column chromatography. Bright ochre, waxy solid **3BTrz**, 23% yield. TLC: R_f_ = 0.56 (Tol/Et_2_O 95:5); 0.95 (Tol/Et_2_O 85:15). mp: 88–90 °C (DCM). ^1^H NMR (200 MHz, CDCl_3_) δ (ppm) = 8.20 (3 H, *d*, *J* = 15.8 Hz), 7.57 (6 H, *d*, *J* = 8.8 Hz), 6.93 (3 H, *d*, *J* = 15.7 Hz), 6.65 (6 H, *d*, *J* = 8.8 Hz), 3.34 (12 H, *t*, *J* = 7.4 Hz), 1.48–1.73 (12 H, *m*), 1.39 (12 H, *qd*, *J* = 7.2 and 14.7 Hz), 0.99 (18 H, *t*, *J* = 7.2 Hz). ^13^C-NMR (50 MHz, CDCl_3_) δ (ppm) = 171.4, 149.3, 141.4, 130.0, 122.8, 120.9, 111.4, 50.8, 29.5, 20.4, 14.0. Anal. calcd for C_51_H_72_N_6_: C 79.64, H 9.44, N 10.93; found: C 78.88, H 9.46, N 10.65.

### Characterization

^1^H-NMR (200 MHz) and ^13^C-NMR (50 MHz) spectra were measured with a BRUKER AC-E 200 FT-NMR-spectrometer. The chemical shift (s = singlet, bs = broad singlet, d = doublet, t = triplet, m = multiplet) is displayed in ppm using the non deuterated solvent as internal standard. Solvents with a grade of deuteration of at least 99.5% were used and purchased at EURISOTOP. NMR-spectra of the aminostyryl-1,3,5-triazines are provided in the Supplementary information. Melting points were measured with the aid of an automated melting point system (SRS OptiMelt). HR-MS analysis was performed on an Agilent 6230 LC TOFMS mass spectrometer equipped with Agilent Dual AJS ESI-Source. Elemental microanalysis was carried out with an EA1108 CHNS-O analyzer from Carlo Erba at the microanalytical laboratory of the Institute for Physical Chemistry at the University of Vienna.

### UV/Vis-spectroscopy

Absorption spectra were measured on a Cary 50 spectrometer, whereas emission spectra were recorded on a FluoroMax-4 (Horiba Scientific). All emission spectra were corrected for the wavelength dependent sensitivity of the detector using a set of secondary emissive standards^29^. Emission quantum yields were obtained using coumarin 30 and coumarin 153 in MeCN^30,31^, as well as rhodamine 6G in methanol as reference standards^32^.

### Open aperture z-scan

An amplified Ti:sapphire laser system (Femtopower Compact Pro) was used for the open aperture z-scan measurement to determine 2PA cross sections. A detailed description of the experimental setup and the fitting equations used can be found elsewhere^33^. Rhodamine B in MeOH was used as reference to verify the reproducibility of the measurements. All investigated compounds were prepared as 10 mM solutions in spectroscopic grade THF. The solutions were measured in a 0.2 mm thick flow cell in a non-recycling volumetric flow of 4 mL/h. The excited volume is therefore refreshed approximately every 100 pulses, which approximately corresponds to 10 times for each z-position, which was found to be sufficient. The measurements were carried out at different pulse energies. At higher energies a signal of the pure solvent appears and the solvent will contribute to the effective nonlinear absorption and even thermal effects are more likely to influence the measurement. Care had to be taken to collect the whole transmitted laser energy using a large diameter and short focal length lens. Additionally, a proper Gaussian beam profile in time and space is essential for the analysis.

### 2PEF-cross section measurements

2PA cross sections were also determined via two-photon excitation spectra using a set-up similar to the one described in literature by Makarov *et al*.^34^. In detail, the output of an optical parametric amplifier (TOPAS-Prime, Light Conversion), which is seeded by a femtosecond Ti:Sapphire regenerative amplifier (Spitfire, Spectra Physics), in combination with a frequency mixer unit (NirUVis, Light Conversion) is used as excitation source. The excitation intensity is adjusted using a combination of a broadband zero-order halfwaveplate and a Glan-Taylor polarizer, and the polarization set to vertical. The beam is slightly focused by a lens (*f* = 20 cm), which is placed 14 cm before the sample. The pump power is monitored using a powermeter (Thorlabs PM100A) equipped with a thermal sensor (Thorlabs S302C) behind the sample. The fluorescence is focused onto the entrance slit of a monochromator (0.25 m Cornerstone, Oriel, grating 74166 Newport) equipped with a multi-pixel photon-counter avalanche photodiode detector (Hamamatsu S-10362-11-050U) using a spherical mirror (Ø = 75 mm, *f* = 150 mm). The output signal is preamplified (SR240, Stanford Research Systems), processed with a gated boxcar-integrator and averager module (SR250, SRS), digitized (SR245, SRS) and recorded on a computer.

The two-photon cross-section at a given wavenumber, σ_2PA_($\,\tilde{\nu }$), was calculated as follows^34^:

$${\sigma }_{2{\rm{P}}{\rm{A}}}(\mathop{\nu }\limits^{ \sim })=\frac{{I}_{{\rm{s}}}(\mathop{\nu }\limits^{ \sim },{\lambda }_{{\rm{o}}{\rm{b}}{\rm{s}},{\rm{s}}})\cdot {c}_{{\rm{r}}}\cdot {\varphi ^{\prime} }_{{\rm{r}}}({\lambda }_{{\rm{o}}{\rm{b}}{\rm{s}},{\rm{r}}})}{{I}_{{\rm{r}}}(\mathop{\nu }\limits^{ \sim },{\lambda }_{{\rm{o}}{\rm{b}}{\rm{s}},{\rm{r}}})\cdot {c}_{{\rm{s}}}\cdot {\varphi ^{\prime} }_{{\rm{s}}}({\lambda }_{{\rm{o}}{\rm{b}}{\rm{s}},{\rm{s}}})}{\sigma }_{{\rm{r}}}^{(2)}(\mathop{\nu }\limits^{ \sim })$$

Here *I*_*x*_($\tilde{\nu }$, λ_obs,*x*_) is the (two-photon-induced) fluorescence intensity at excitation wavenumber $\tilde{\nu }$ and observation wavelength λ_obs_ for either sample or reference (*x* ∈ {s,r}). *c*_*x*_ and *ϕ*_*x*_′(λ_obs_) are the concentration and differential fluorescence quantum yield (at the corresponding observation wavelengths) of sample and reference.

The 2PA spectra were measured relative to rhodamine 6G in methanol and fluorescein in water at pH 11^35^.

Solutions of the investigated compounds were prepared in THF with concentrations in the range from 3∙10^−6^ to 2∙10^−5^ mol/L (the maximum low energy absorption was approx. 0.5). Pump pulse energies were in the range from 1 to 3 µJ.

### Computational Details

Geometry optimizations were performed at the DFT/M06-2X^21^ level of theory using the 6–31 + G* basis set^36^. Solvent effects were included through the conductor-like polarizable continuum model^37^ using values of 7.4257 and 1.971216 for the static and optical dielectric constants, respectively. Excitation energies were computed using the ab-initio algebraic diagrammatic construction method to second order ADC(2)^22,23^. Two-photon absorption strengths were evaluated by a sum-over-states expression evaluated in the intermediate state representation^38–40^. Excited state solvation was included by a perturbative linear-response approach in the nonequilibrium limit^41^. All calculations were carried out employing the Q-Chem 4.3 program package^42^.

### 2PP structuring tests

The details of the 2PP microfabrication setup were reported previously^43^. For the present work a tunable femtosecond NIR-laser (MaiTai eHP DeepSee, Spectra-Physics) was used at various wavelengths, with a pulse duration of 70 fs after the microscope objective (32×/0.85, water immersion) used to focus the beam into the sample. The prism pulse compressor position was optimized for every wavelength to ensure a 70 fs pulse in the sample. The peak intensities for the used objective and 1 mW of laser power for a sech² shaped pulse are calculated according to Zipfel *et al*.^44^ - 750 nm: 63.45 GW/cm², 760 nm: 61.8 GW/cm², 800 nm: 55.77 GW/cm², 815 nm: 53.74 GW/cm². For a 32×/0.85 microscope objective, the size of the focal point can be calculated to FWHM_xy_ = 0.35 µm and FWHM_z_ = 1.77 µm at 800 nm by using the formulas provided by Zipfel *et al*.^44^. This corresponds to approximately 571,000 voxel/s at 200 mm/s. By using a line and layer spacing of half the FWHM the throughput of the system can be estimated with 0.11 mm³/h. To facilitate high-speed structuring a combination of sample positioning via a motorized stage and a galvanometer scanner was used. The in-house developed software controls the complete setup. The structuring process was monitored in real time with a CMOS-camera mounted behind the dichroic mirror in the beam path. The samples for 2PP tests were prepared by casting about 30 µL of a liquid formulation (2PI dissolved at concentrations of 3.3 µmol 2PI/g resin (**3BTrz**), 5 µmol 2PI/g resin (**M2CMK**, **2BTrz**) or 10 µmol 2PI/g resin (**BTrz**) in a 1:1 mixture of trimethylolpropane triacrylate (TTA, Genomer 1330) and ethoxylated-(20/3)-trimethylolpropane triacrylate (ETA, Sartomer 415), **3BTrz** and **M2CMK** had to be pre-dissolved in acetone that was removed in vacuo before processing) onto the glass substrate of a µ-dish (35 mm diameter with glass bottom, high version, Ibidi GmbH, Martinsried, Germany) that had been functionalized with methacrylate groups by cleaning and activation with a 4:1 mixture of conc. H_2_SO_4_ and H_2_O_2_ (30% in water), then using 3-(trimethoxysilyl)propyl methacrylate (Sigma Aldrich) according to literature^45^. Arrays of defined woodpile test structures (lateral dimension: 30 × 30 µm, 3 µm hatch-distance, 0.5 µm layer-distance, 25 layers) were written into the monomer formulation by means of 2PP. After polymerization, the unpolymerized resin was removed by repeated soaking in Isopropanol. For time efficient visual assessment of structure quality, laser scanning microscopy (Zeiss LSM 700 and ZEN11 software for evaluation, detailed images of the structures are provided in the Supplementary information) imaging was employed, taking advantage of the autofluorescence of residual 2PIs in the polymer structures and averaging the signal from stacks of multiple scanned layers to visualize internal defects of structures that would remain hidden in electron microscopy images. The complex polyhedron model was obtained analogous to the woodpile arrays at 100 mW power and 200 mm/s writing speed, with the rinsed structure dried, sputtered with gold and images taken using a FEI Philips XL30 scanning electron microscope with EDX detection.

## Electronic supplementary material

Supporting Information
