# Supplementary material for: Wavelength-optimized Two-Photon Polymerization Using Initiators Based on Multipolar Aminostyryl-1,3,5-triazines
Source: Sci Rep. 2018 Nov 22;8:17273. doi: 10.1038/s41598-018-35301-x (PMC6250671; doi:10.1038/s41598-018-35301-x)

Supporting Information for

Wavelength-optimized Two-Photon Polymerization Using Initiators Based on Multipolar Aminostyryl-1,3,5-triazines

Maximilian Tromayer, Peter Gruber, Arnulf Rosspeintner, Aliasghar Ajami, Wolfgang Husinsky, Felix Plasser, Leticia González, Eric Vauthey, Aleksandr Ovsianikov, Robert Liska*

email: robert.liska@tuwien.ac.at

Spectral Data

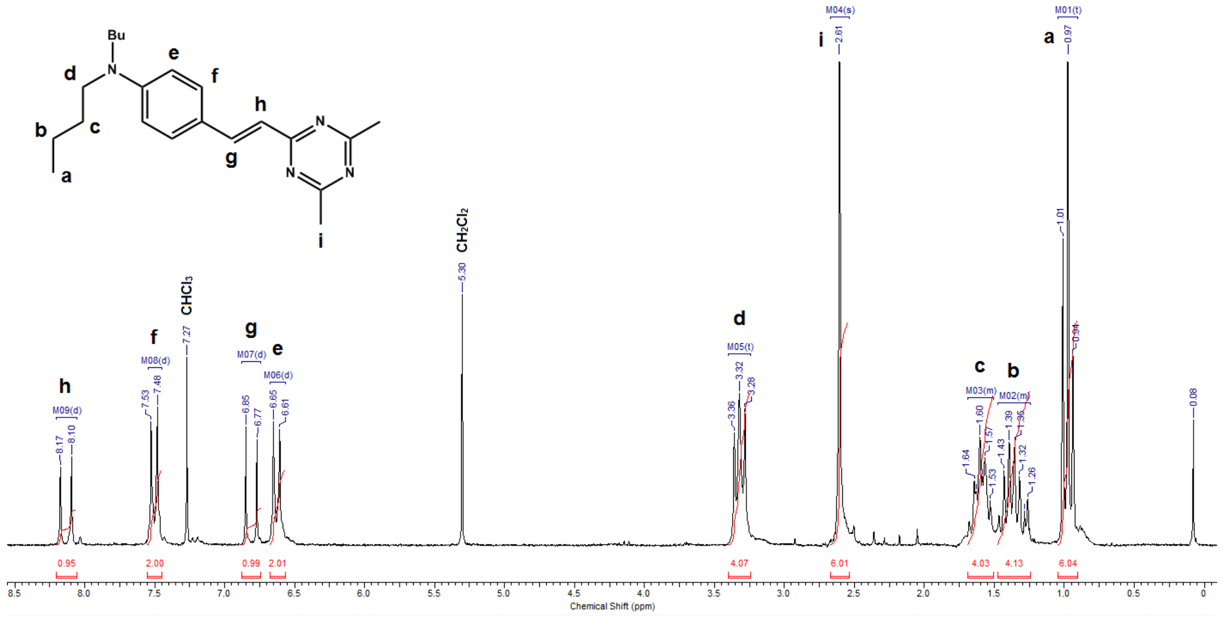

Figure S1. ^{1}H NMR of **BTrz**

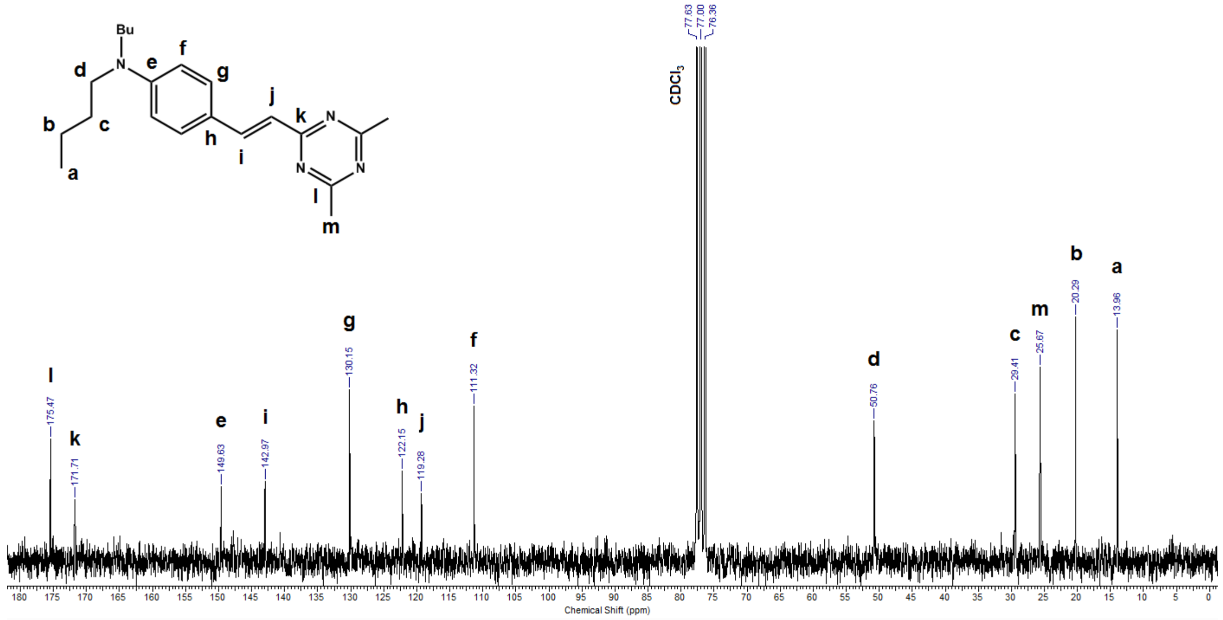

Figure S2. ^{13}C NMR of **BTrz**

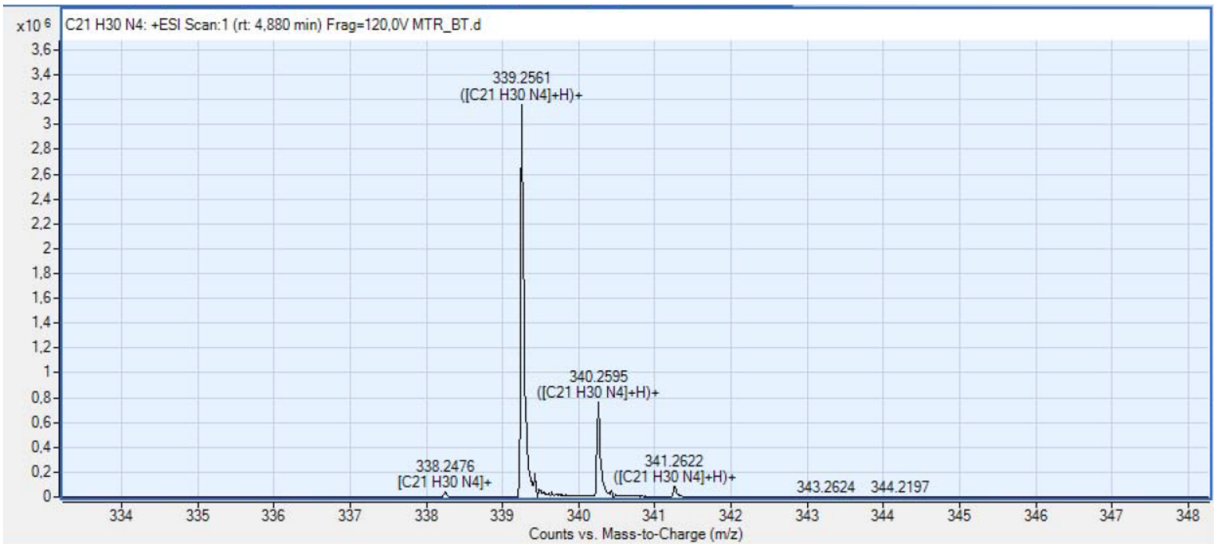

Figure S3. HRMS showing $[M+H]^+$-peak of **BTrz**

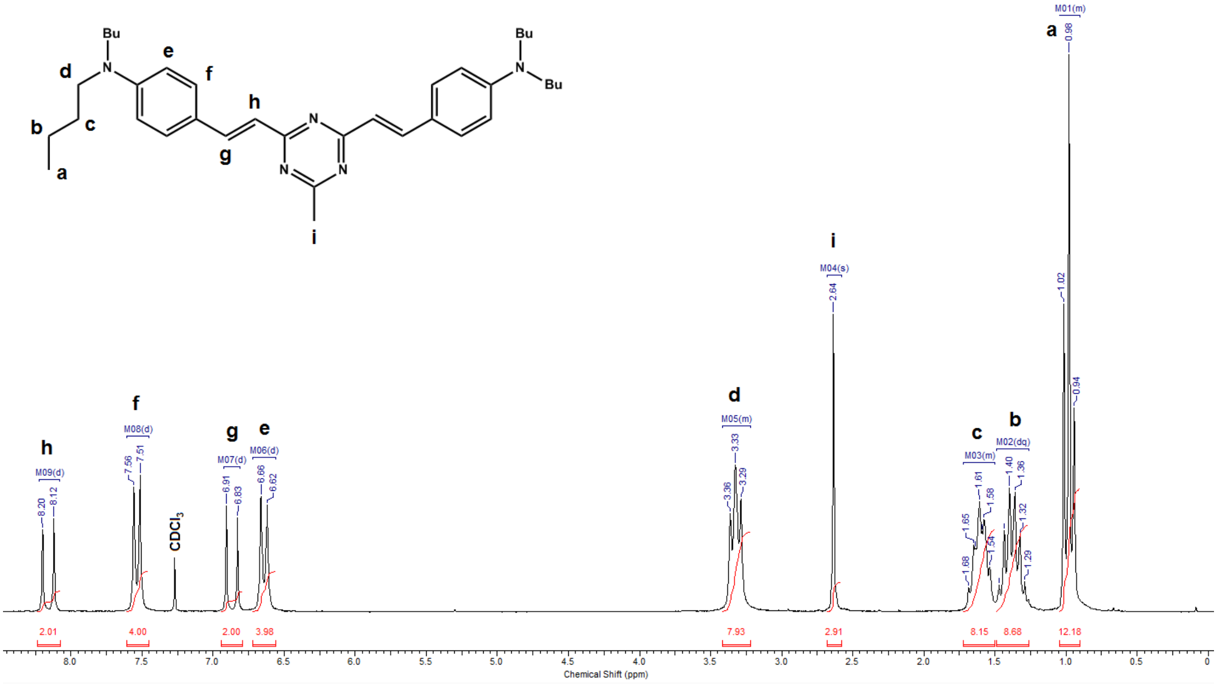

Figure S4. ^{1}H NMR of **2BTrz**

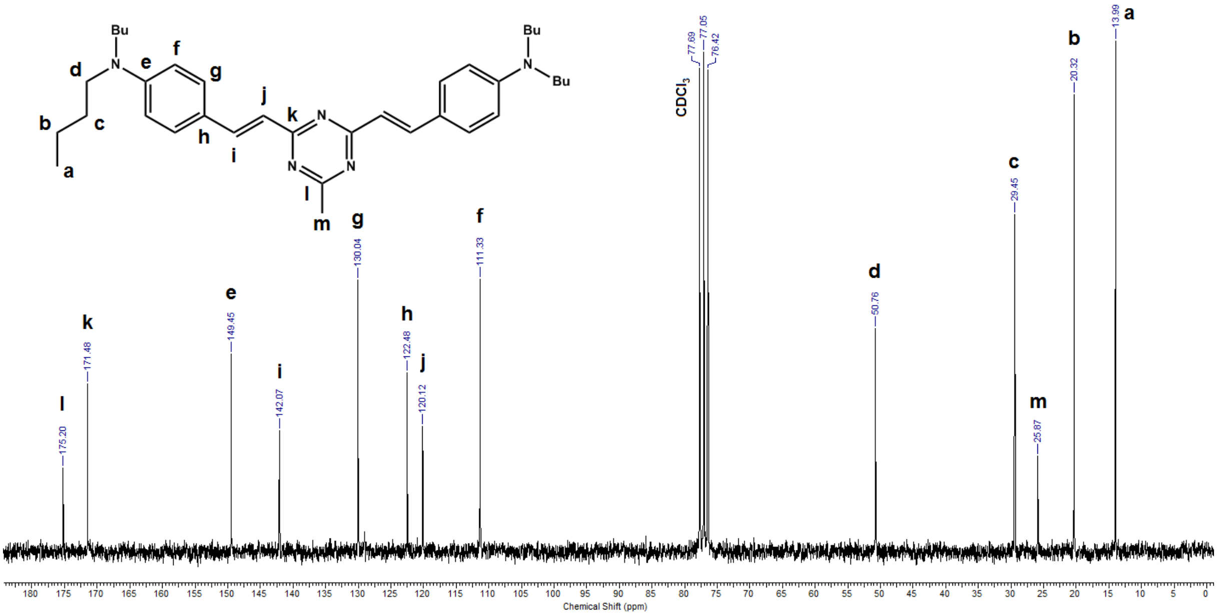

Figure S5. ^{13}C NMR of **2BTrz**

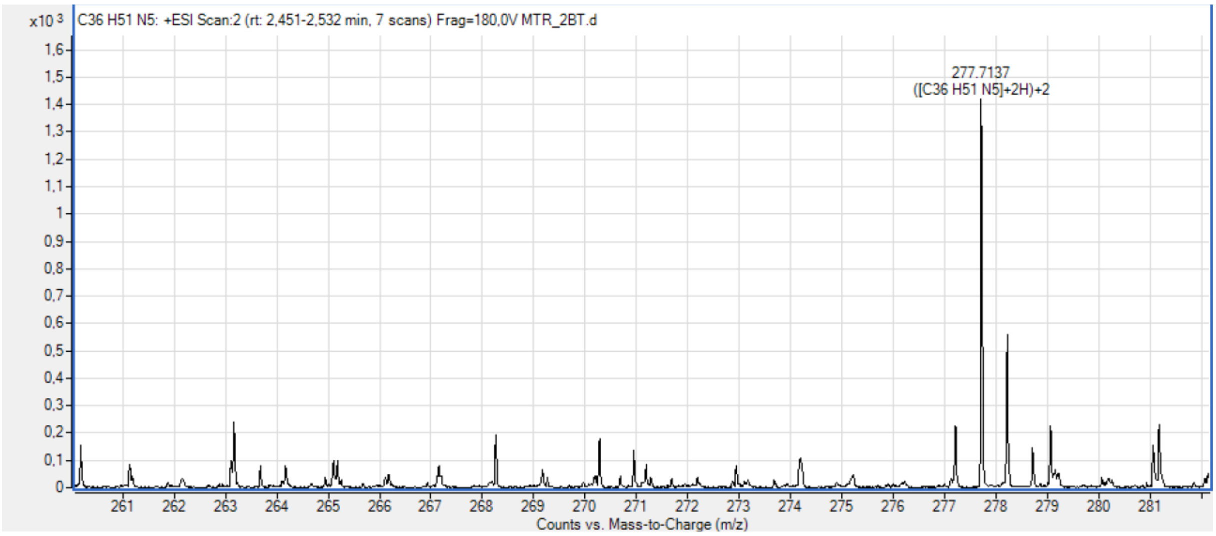

Figure S6. HRMS showing $[M+2H]^{2+}$-peak of **2BTrz**

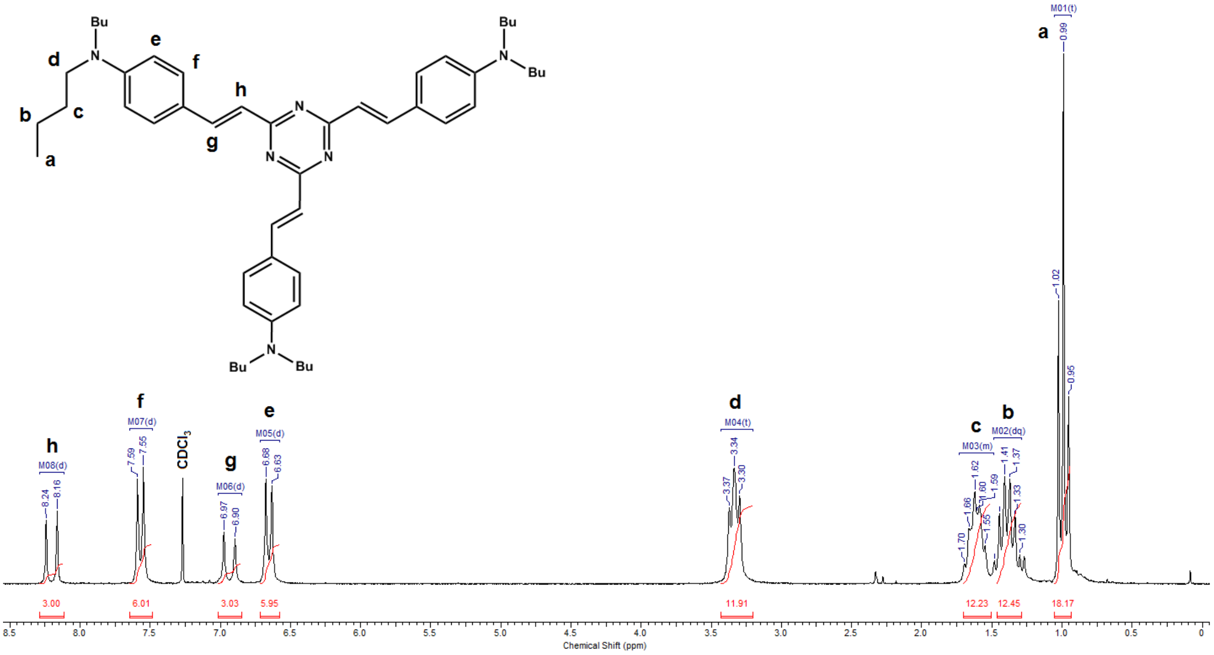

Figure S7. ^{1}H NMR of **3BTrz**

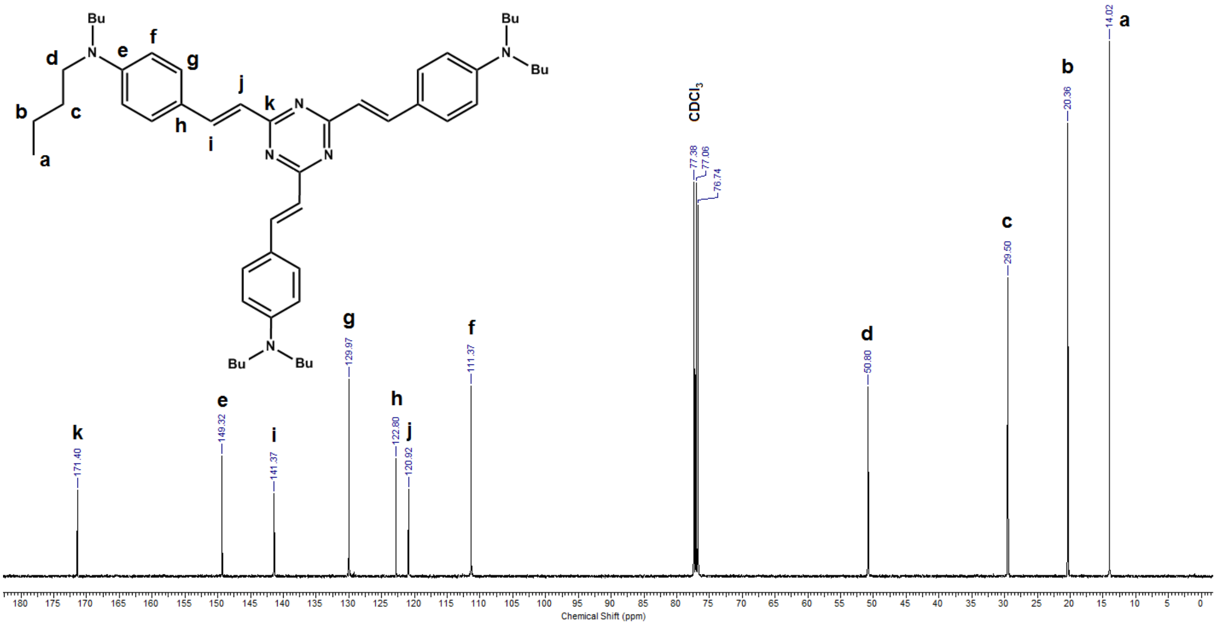

Figure S8. ^{13}C NMR of **3BTrz**

Two-Photon Polymerization Processing Windows

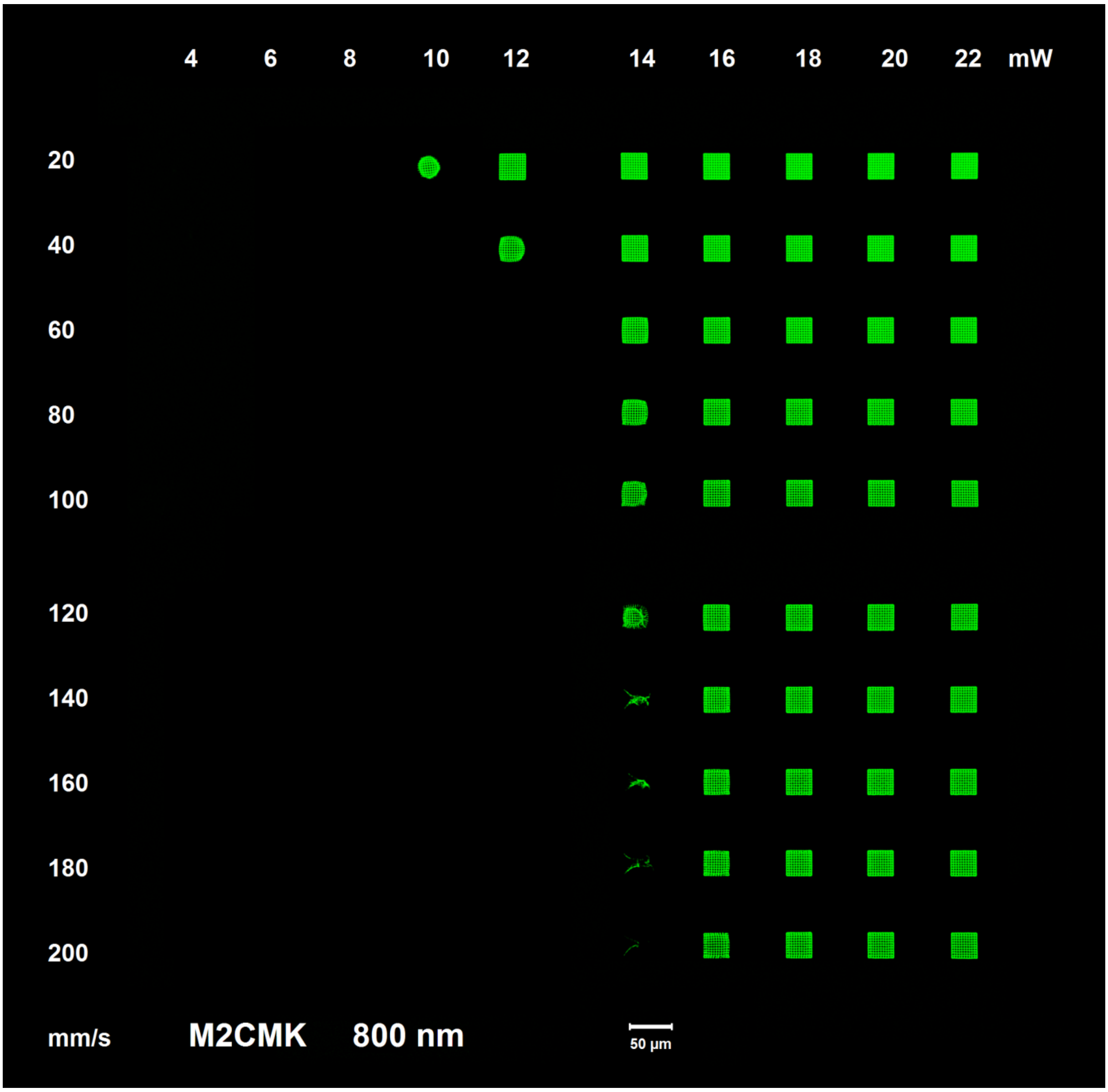

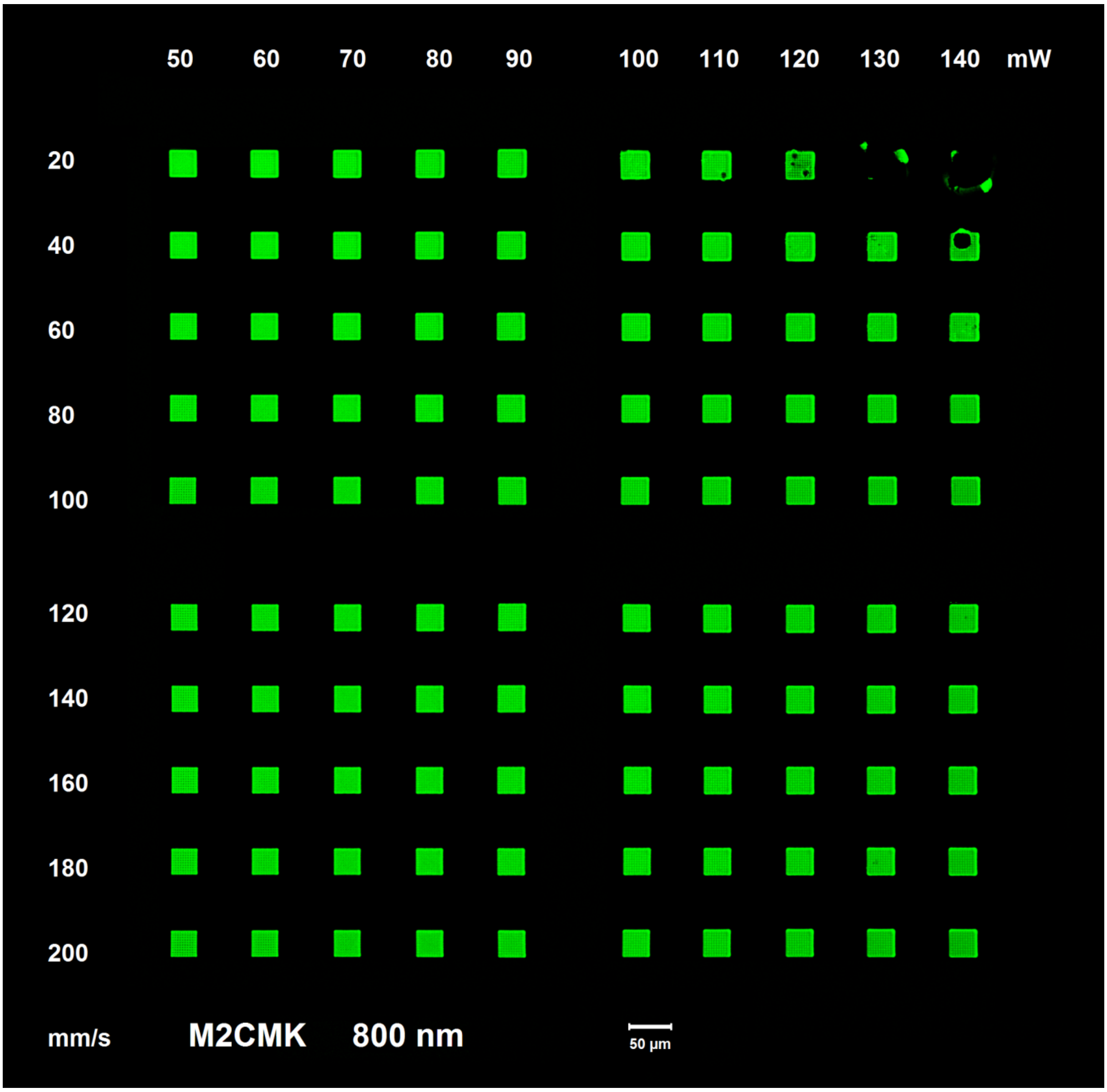
50
60
70
80
90
100
110
120
130
140
mW
20
40
60
80
100
120
140
160
180
200
mm/s
M2CMK
800 nm
50 µm

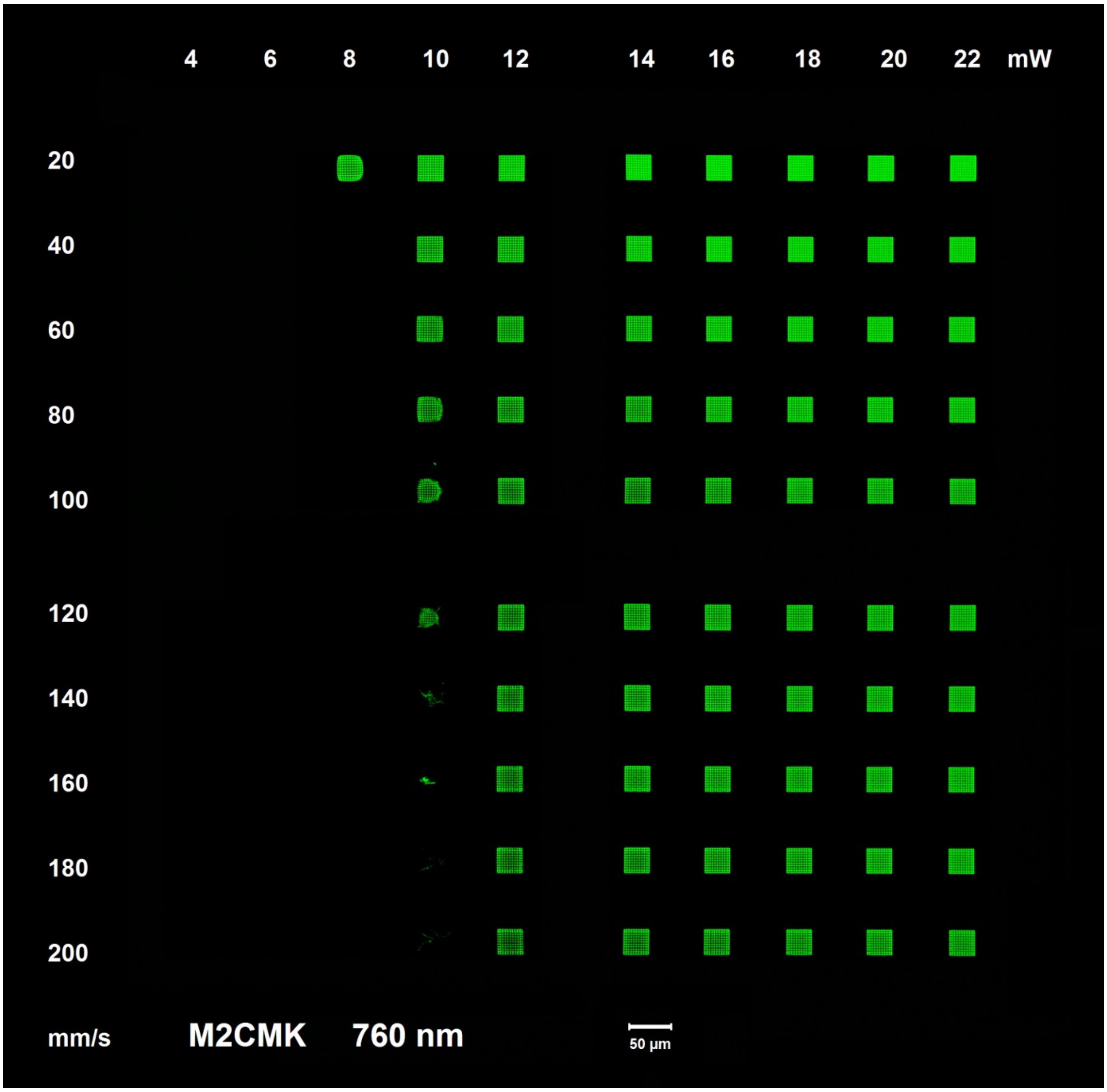
4
6
8
10
12
14
16
18
20
22
mW
20
40
60
80
100
120
140
160
180
200
mm/s
M2CMK
760 nm
50 µm

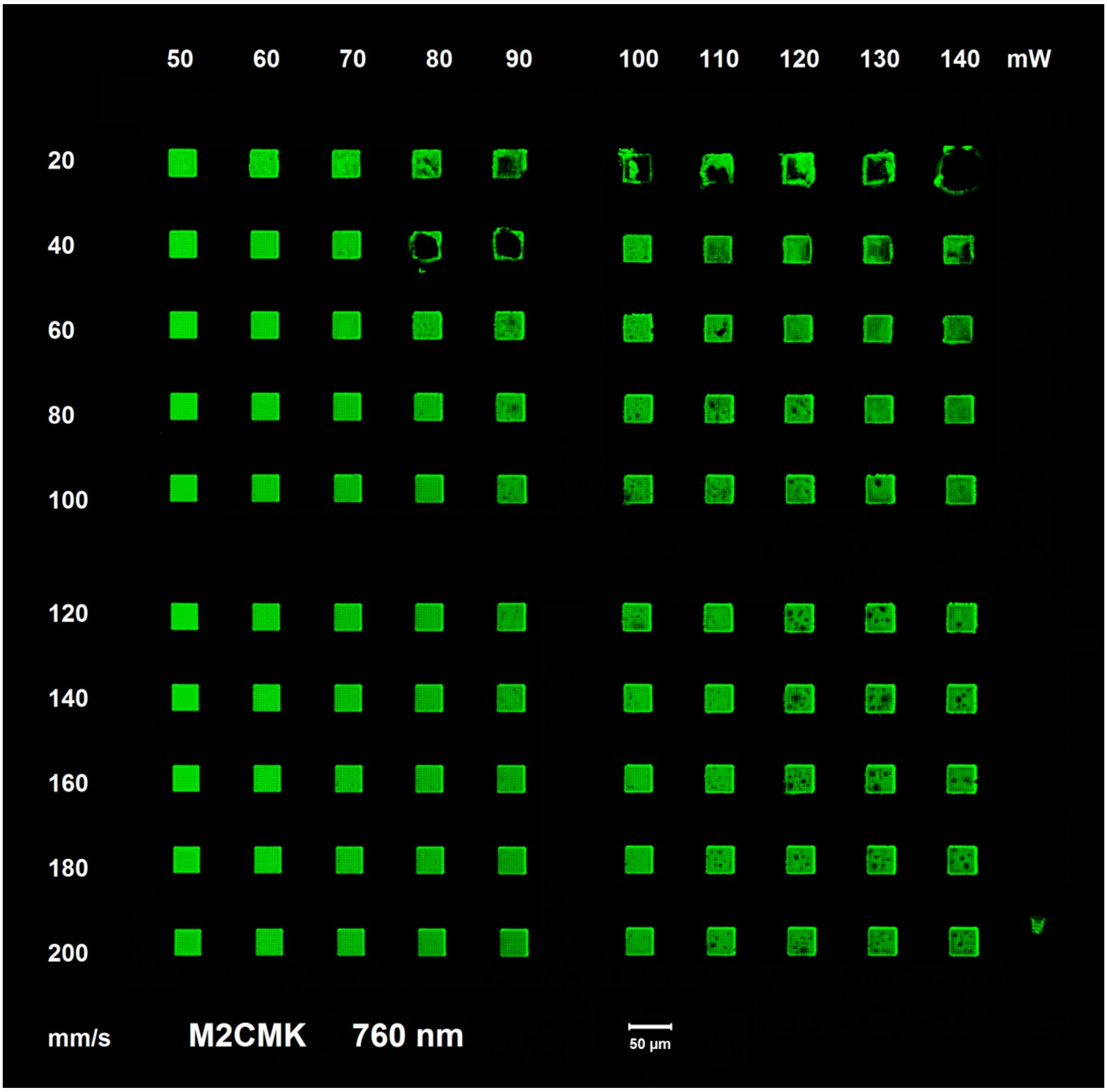

50
60
70
80
90
100
110
120
130
140
mW
20
40
60
80
100
120
140
160
180
200
mm/s
M2CMK
760 nm
50 µm

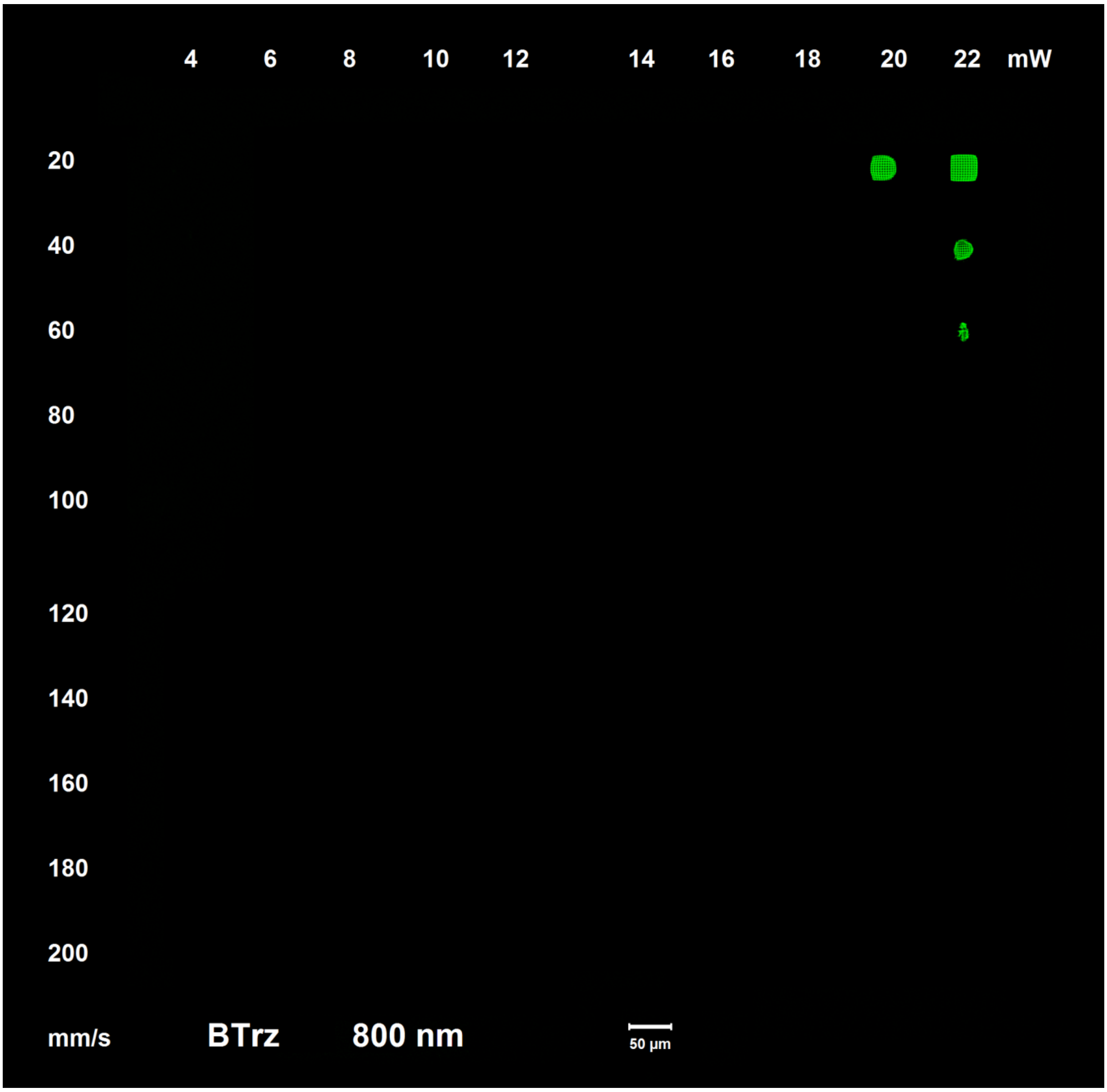
4
6
8
10
12
14
16
18
20
22
mW
20
40
60
80
100
120
140
160
180
200
mm/s
BTrz
800 nm
50 µm

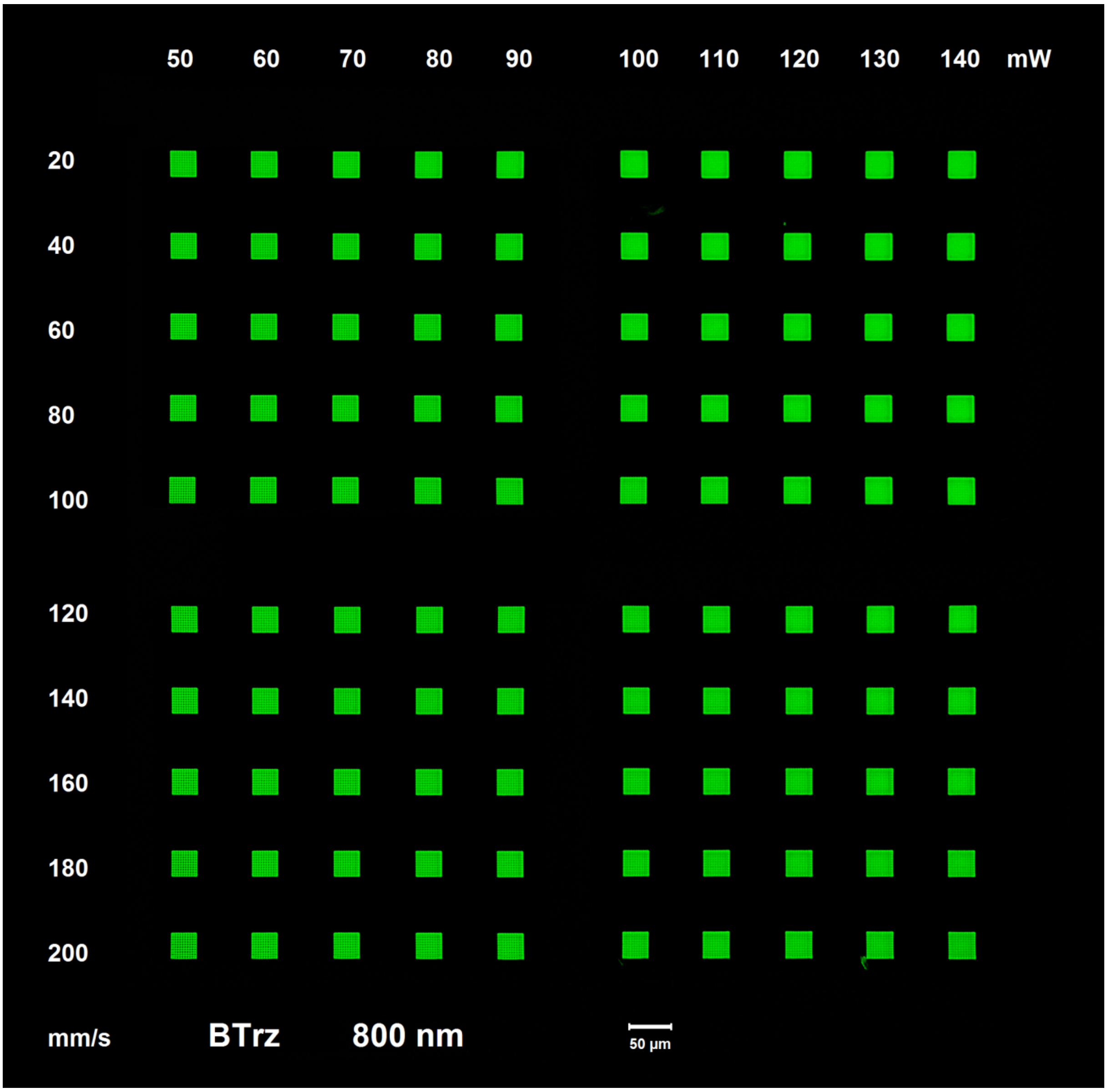
50
60
70
80
90
100
110
120
130
140
mW
20
40
60
80
100
120
140
160
180
200
mm/s
BTrz
800 nm
50 µm

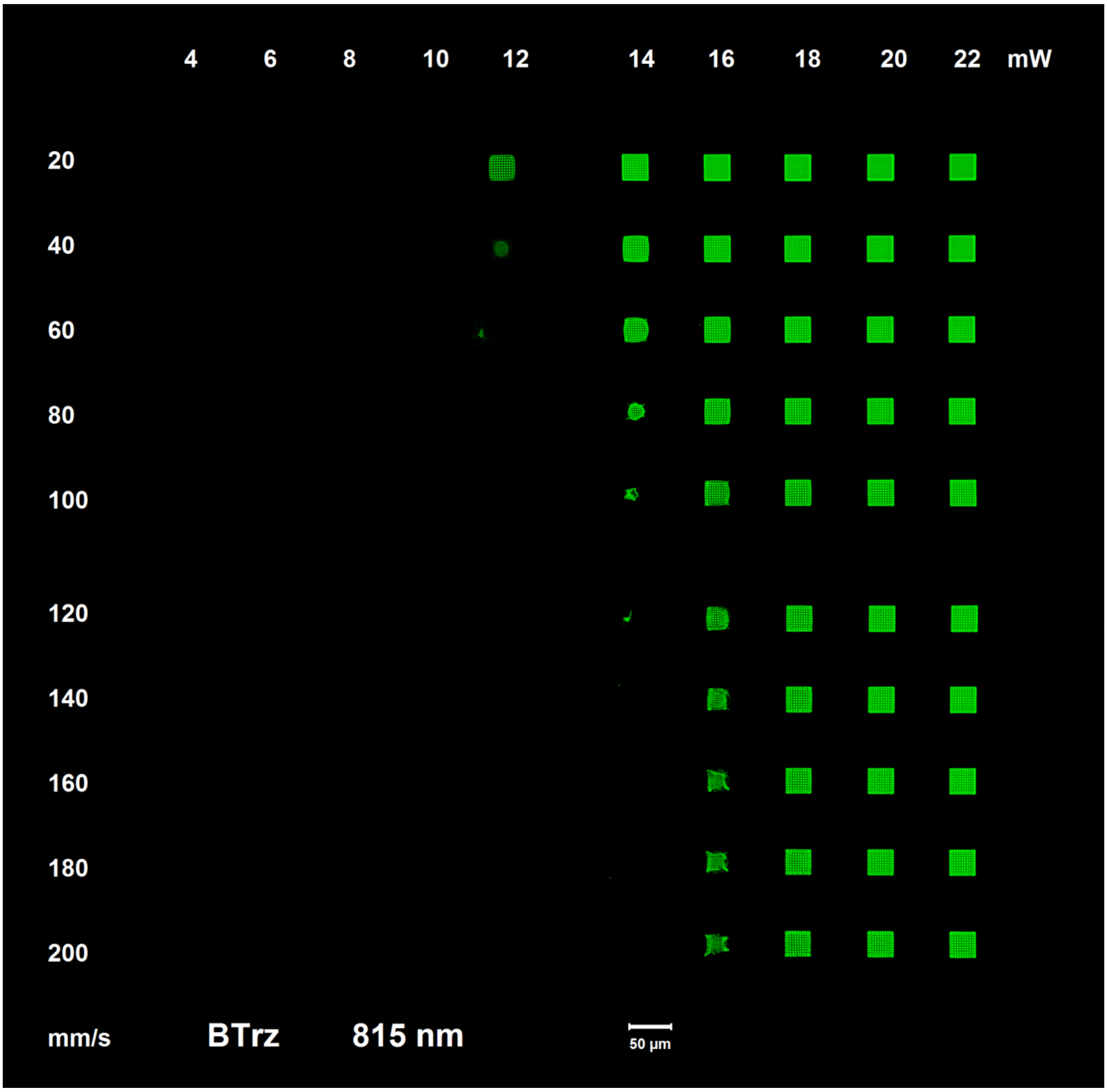

4
6
8
10
12
14
16
18
20
22
mW
20
40
60
80
100
120
140
160
180
200
mm/s
BTrz
815 nm
50 µm

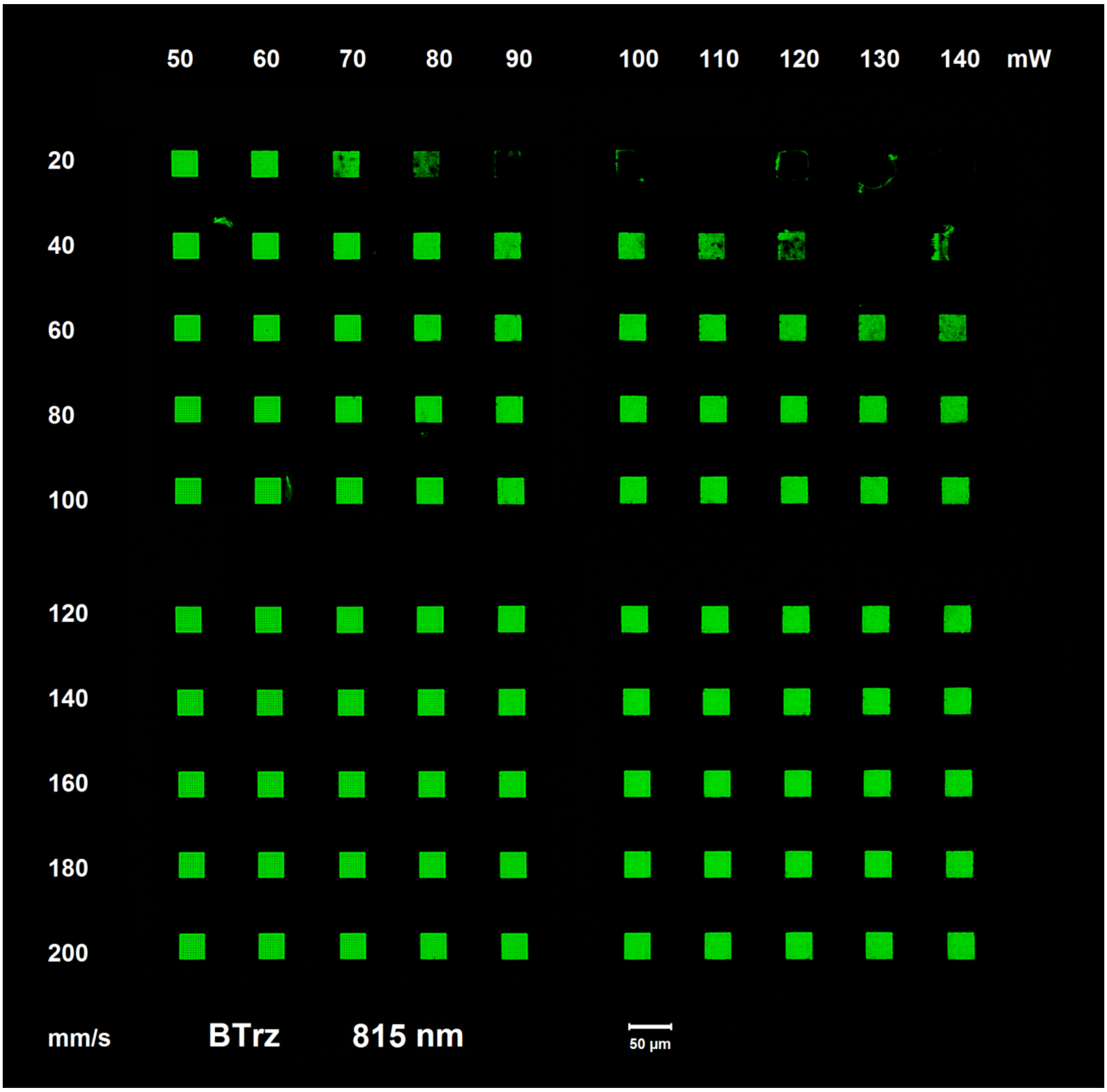
50
60
70
80
90
100
110
120
130
140
mW
20
40
60
80
100
120
140
160
180
200
mm/s
BTrz
815 nm
50 µm

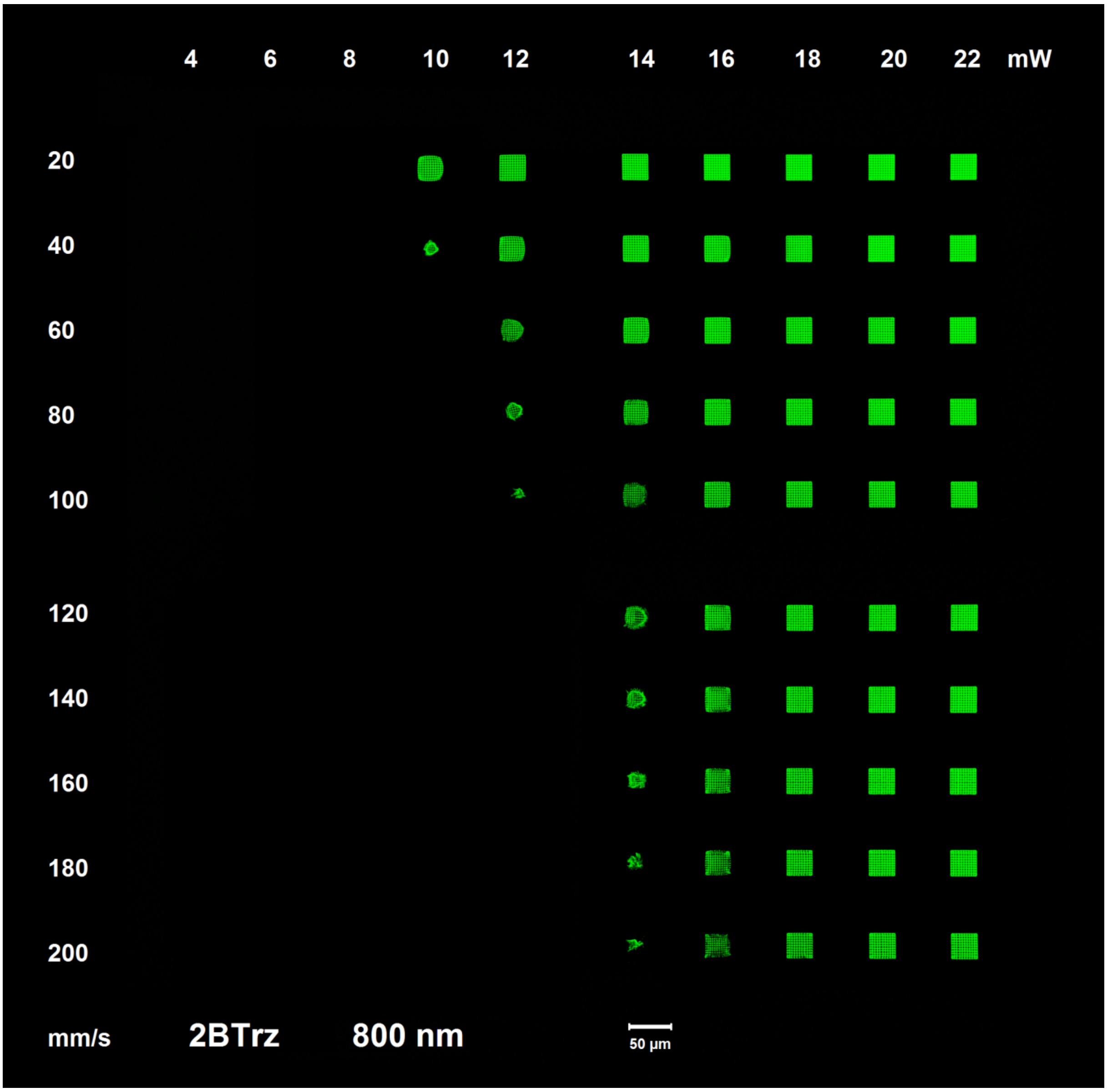
4
6
8
10
12
14
16
18
20
22
mW
20
40
60
80
100
120
140
160
180
200
mm/s
2BTrz
800 nm
50 µm

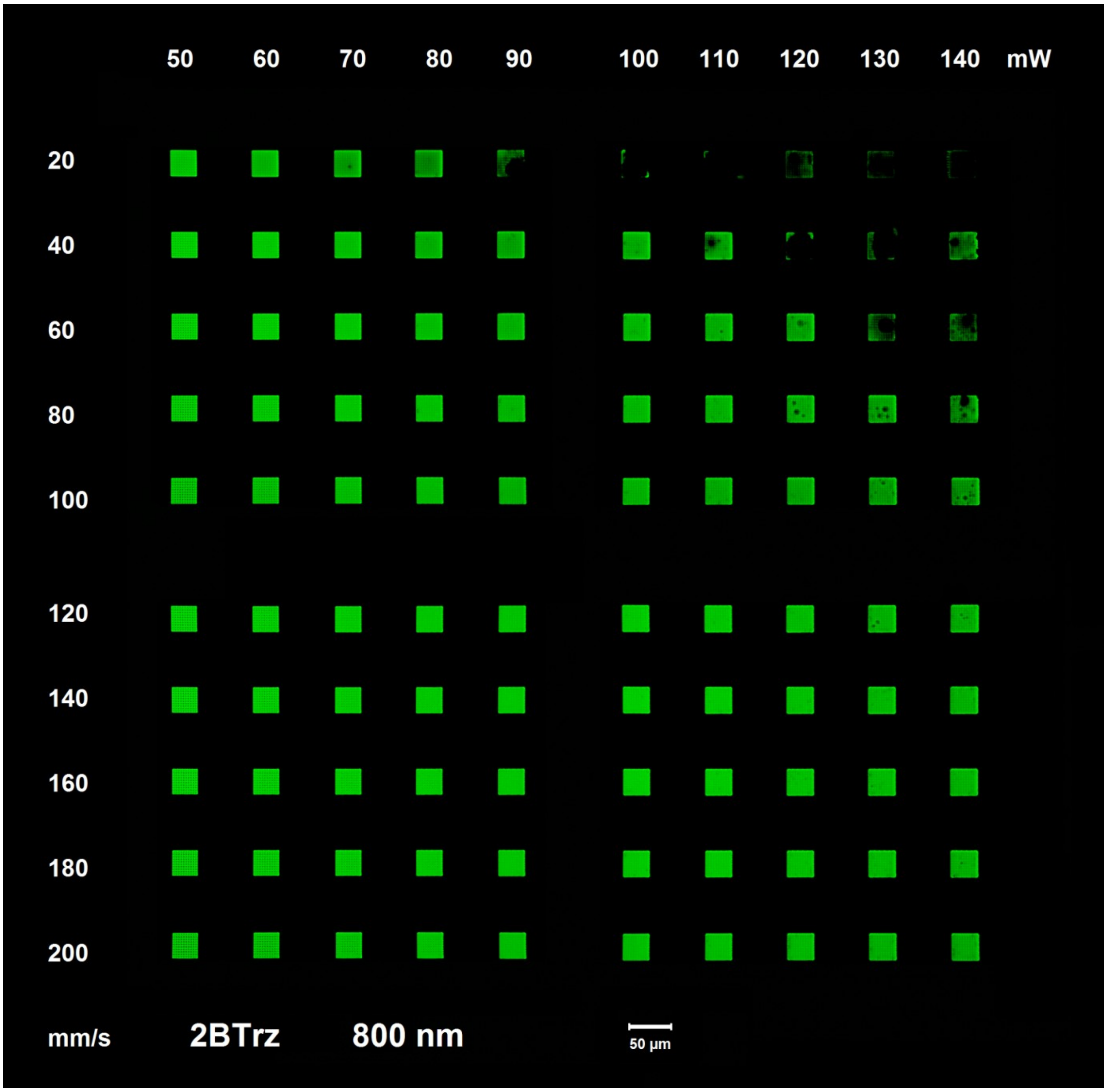
50
60
70
80
90
100
110
120
130
140
mW
20
40
60
80
100
120
140
160
180
200
mm/s
2BTrz
800 nm
50 µm

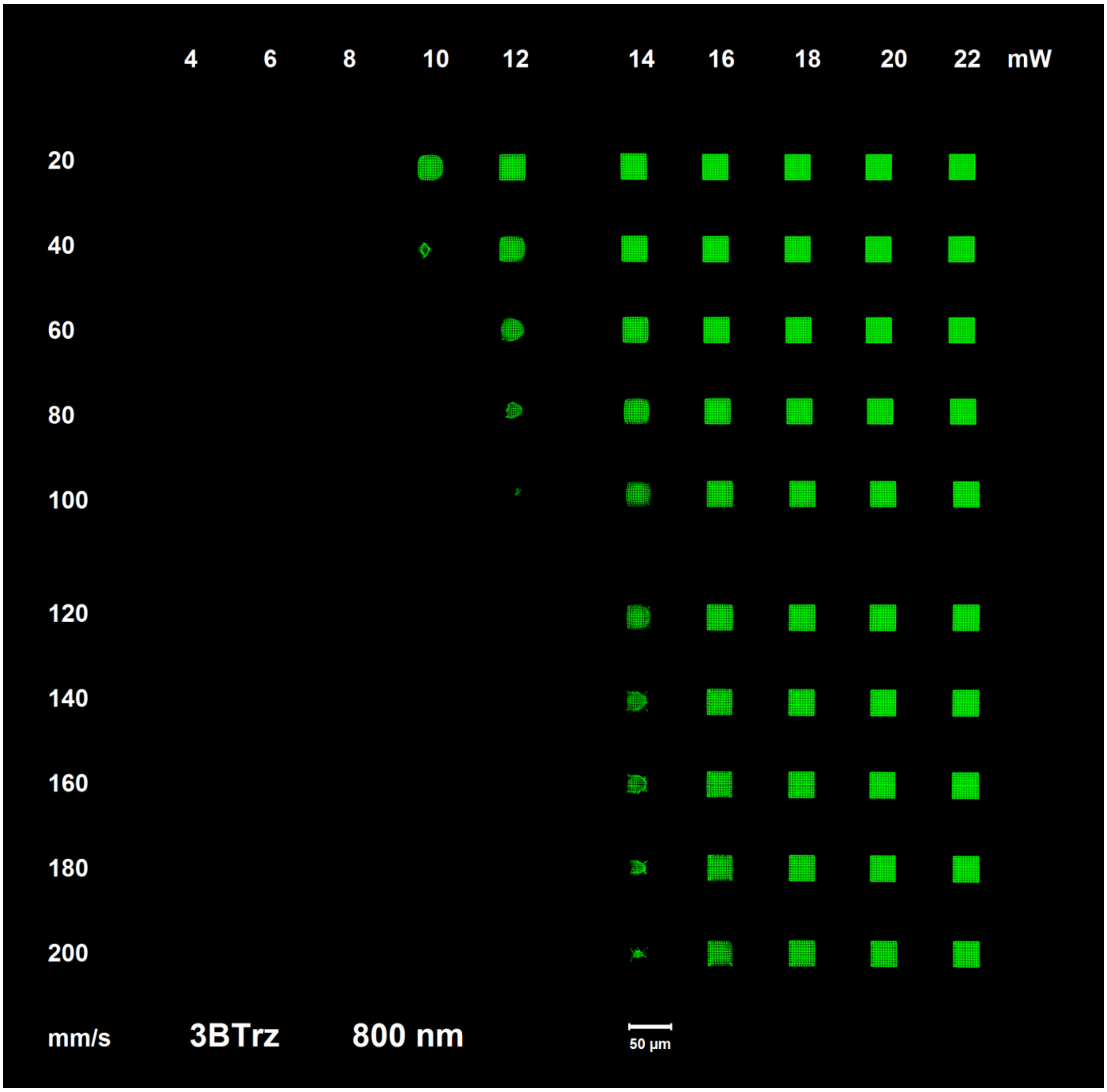
4
6
8
10
12
14
16
18
20
22
mW
20
40
60
80
100
120
140
160
180
200
mm/s
3BTrz
800 nm
50 µm

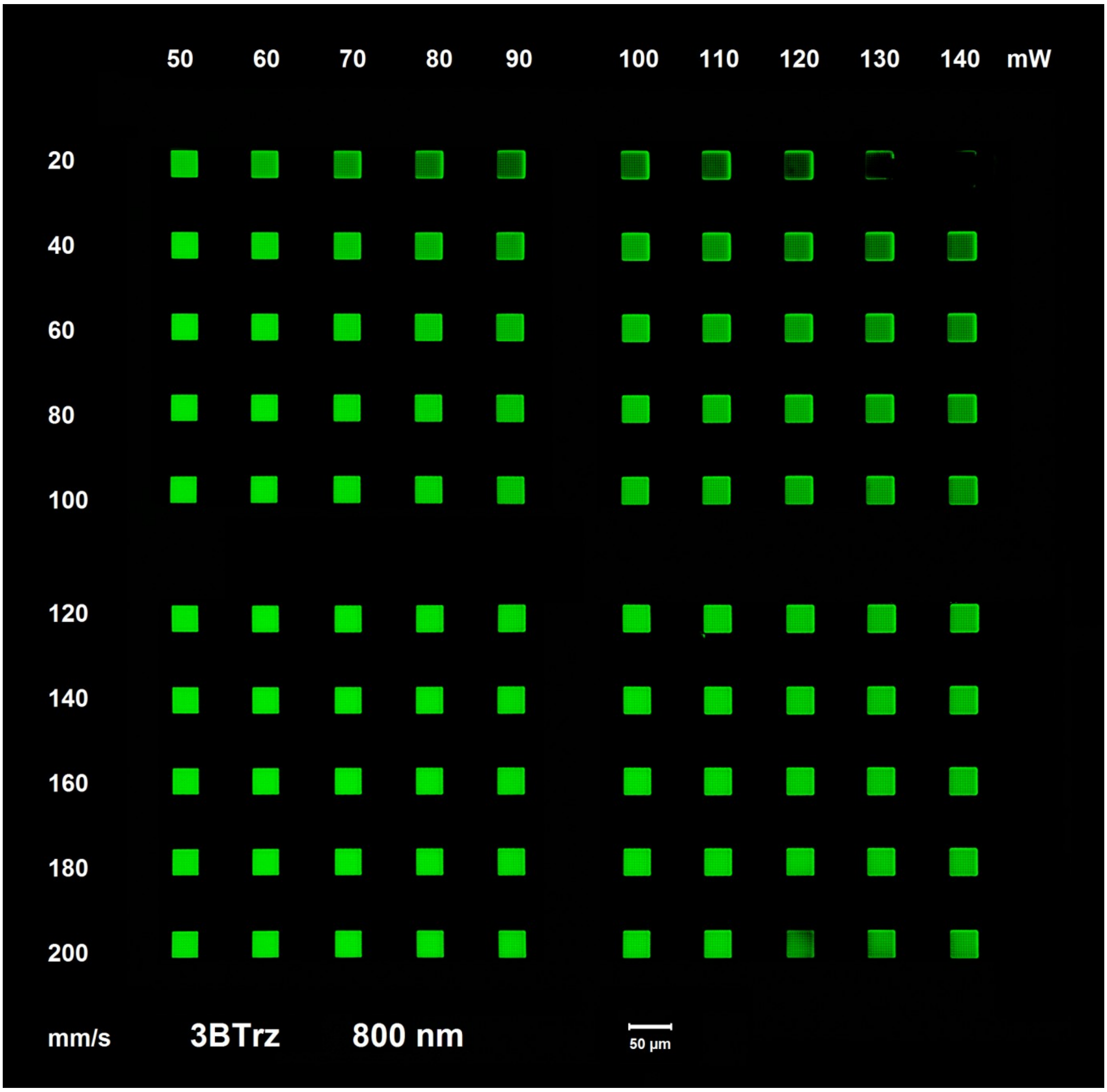
50
60
70
80
90
100
110
120
130
140
mW
20
40
60
80
100
120
140
160
180
200
mm/s
3BTrz
800 nm
50 µm

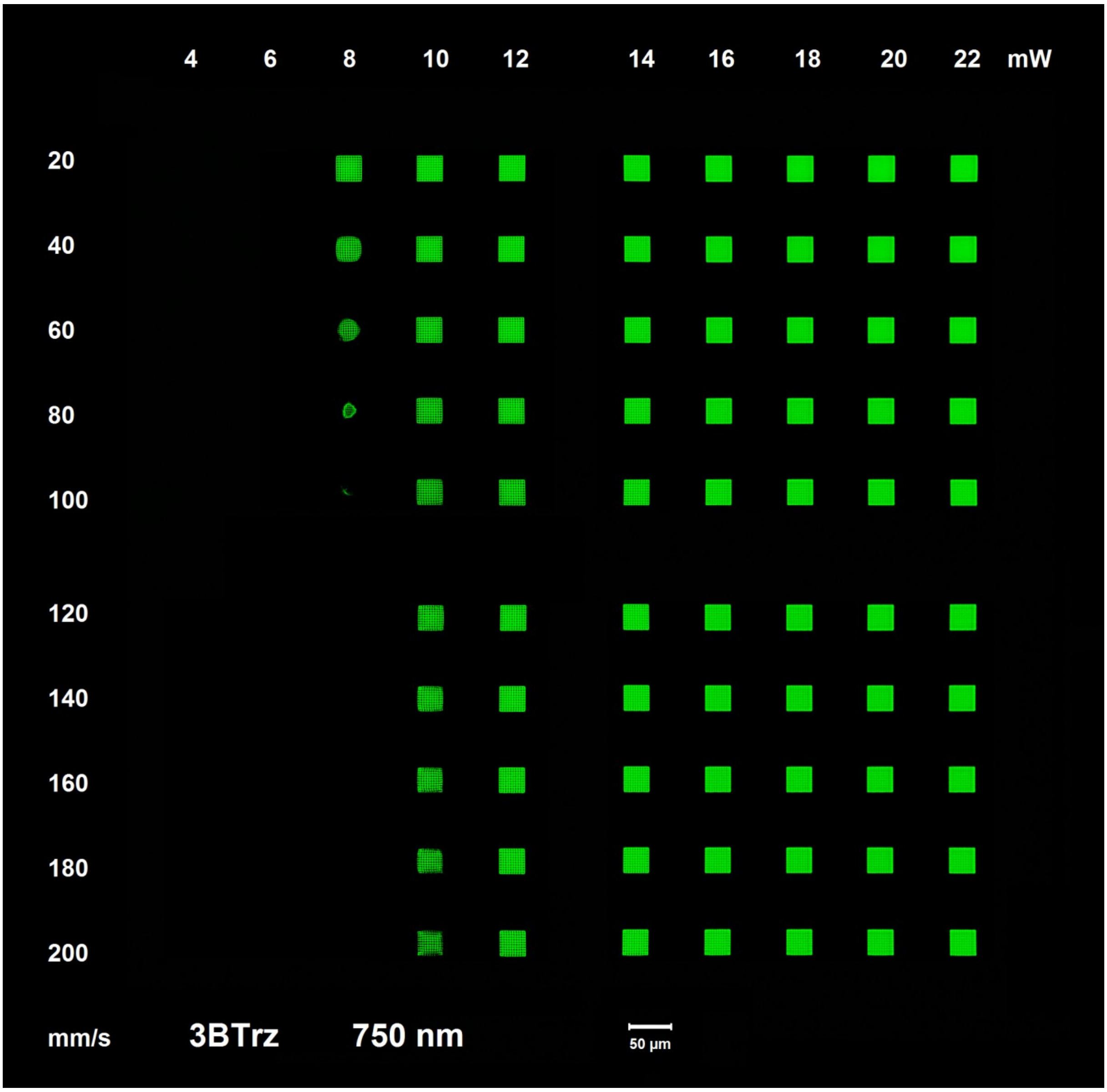
4
6
8
10
12
14
16
18
20
22
mW
20
40
60
80
100
120
140
160
180
200
mm/s
3BTrz
750 nm
50 µm

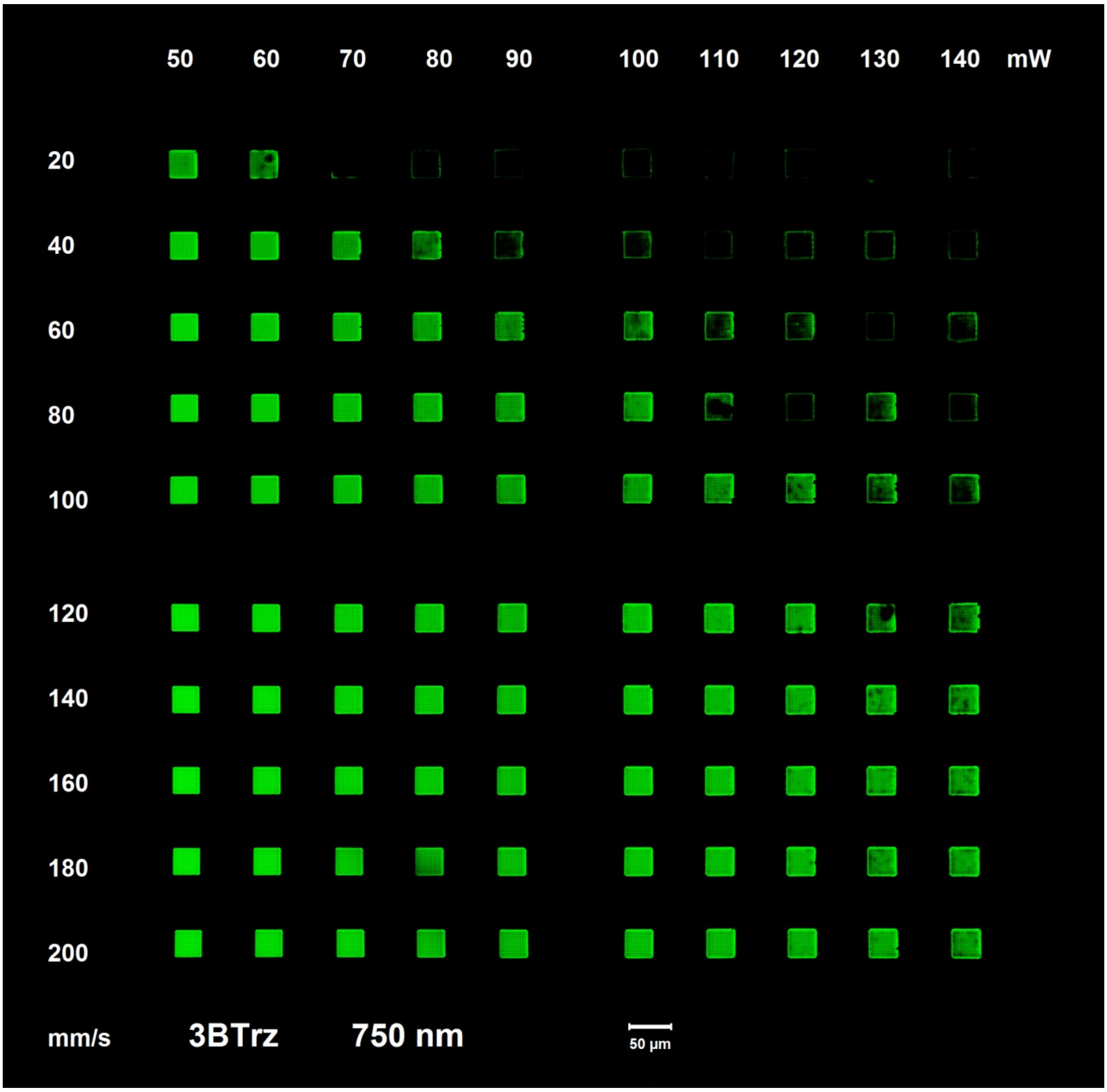
50
60
70
80
90
100
110
120
130
140
mW
20
40
60
80
100
120
140
160
180
200
mm/s
3BTrz
750 nm
50 µm

SEM image of a defect-free woodpile structure

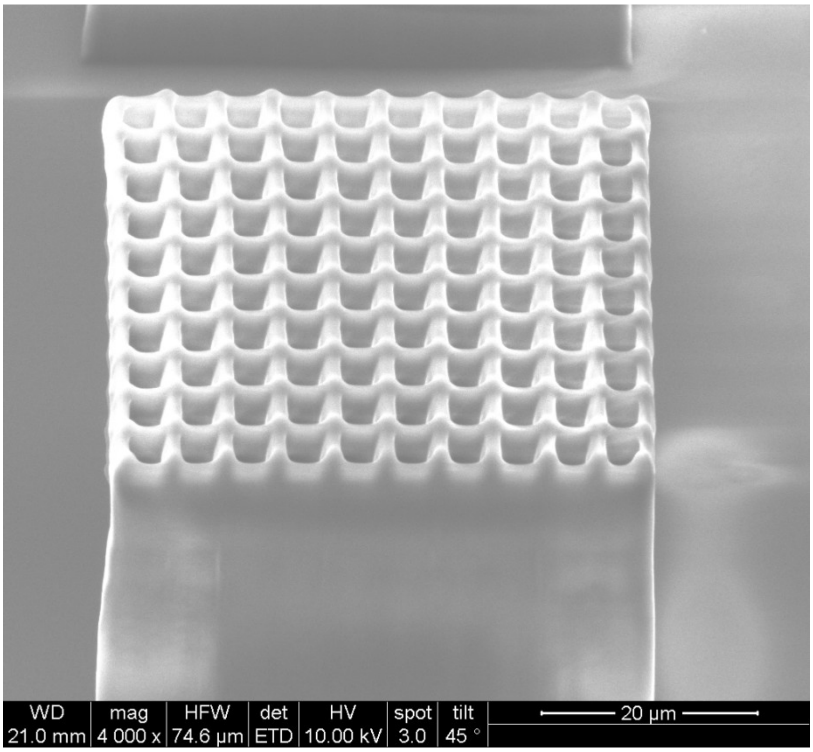

Control Measurements of ETA/TTA 1:1 without photoinitiators

Polymerization of ETA/TTA initiated by 2PA induced avalanche ionization is possible, but polymerization thresholds are high and no defect-free structures could be obtained (light microscopy images).

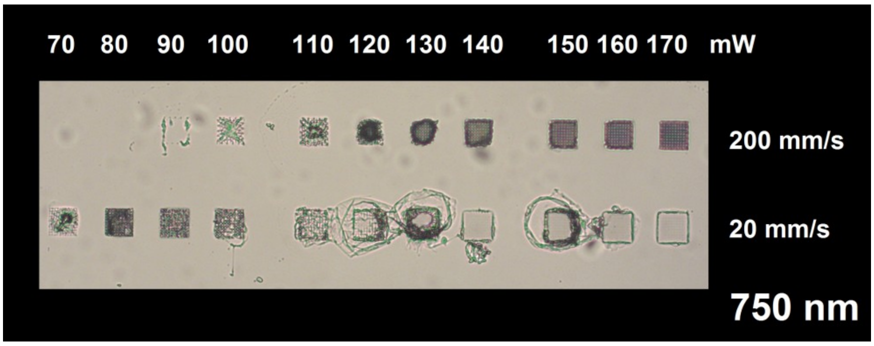

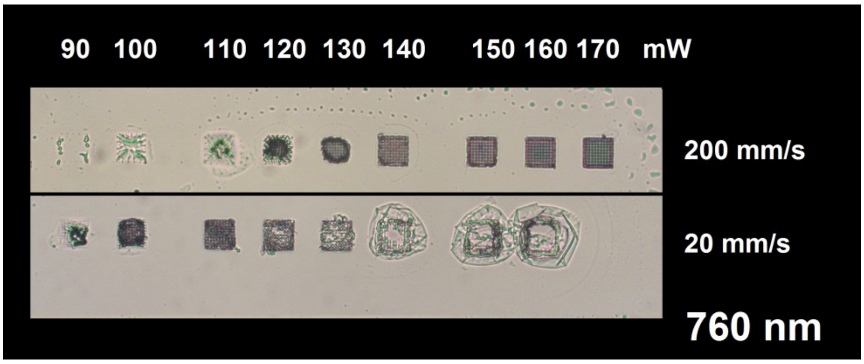

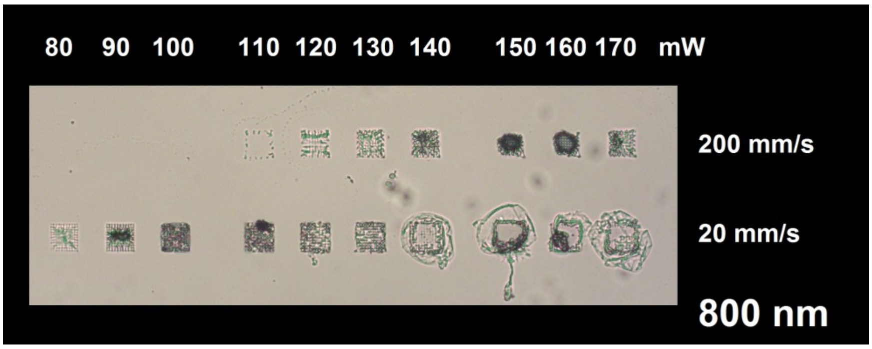

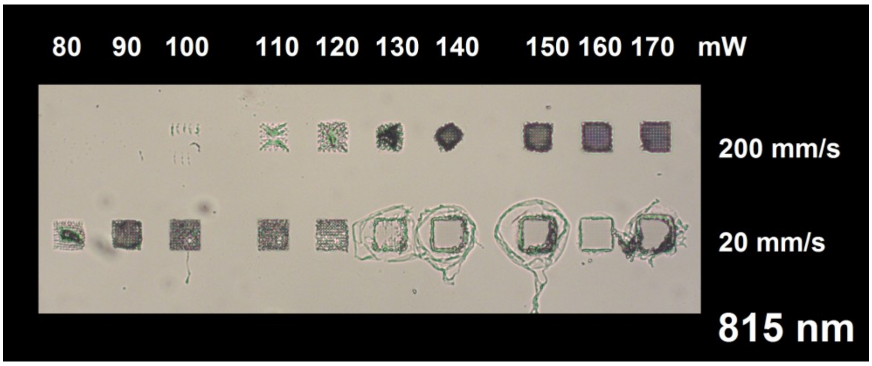

Supplement: Supplementary file 1 — Supporting Information [file 41598_2018_35301_MOESM1_ESM.pdf]
